# A Systematic Review on Quiescent State Research Approaches in *S. cerevisiae*

**DOI:** 10.3390/cells12121608

**Published:** 2023-06-12

**Authors:** Monika Opalek, Hanna Tutaj, Adrian Pirog, Bogna J. Smug, Joanna Rutkowska, Dominika Wloch-Salamon

**Affiliations:** 1 Institute of Environmental Sciences, Faculty of Biology, Jagiellonian University, 30-387 Krakow, Poland; hanna.tutaj@uj.edu.pl (H.T.); adrian.pirog@doctoral.uj.edu.pl (A.P.); joanna.rutkowska@uj.edu.pl (J.R.); dominika.wloch-salamon@uj.edu.pl (D.W.-S.); 2 Malopolska Centre of Biotechnology, Jagiellonian University, 30-387 Krakow, Poland; bogna.smug@uj.edu.pl

**Keywords:** dormancy, quiescence, growth arrest, budding yeast, stationary phase, G0, cell cycle, eukaryotic cell model

## Abstract

Quiescence, the temporary and reversible arrest of cell growth, is a fundamental biological process. However, the lack of standardization in terms of reporting the experimental details of quiescent cells and populations can cause confusion and hinder knowledge transfer. We employ the systematic review methodology to comprehensively analyze the diversity of approaches used to study the quiescent state, focusing on all published research addressing the budding yeast *Saccharomyces cerevisiae*. We group research articles into those that consider all cells comprising the stationary-phase (SP) population as quiescent and those that recognize heterogeneity within the SP by distinguishing phenotypically distinct subpopulations. Furthermore, we investigate the chronological age of the quiescent populations under study and the methods used to induce the quiescent state, such as gradual starvation or abrupt environmental change. We also assess whether the strains used in research are prototrophic or auxotrophic. By combining the above features, we identify 48 possible experimental setups that can be used to study quiescence, which can be misleading when drawing general conclusions. We therefore summarize our review by proposing guidelines and recommendations pertaining to the information included in research articles. We believe that more rigorous reporting on the features of quiescent populations will facilitate knowledge transfer within and between disciplines, thereby stimulating valuable scientific discussion.

## 1. Introduction

Quiescence is a fundamental biological state of reversible growth arrest in which cells reduce metabolic activity and halt growth, but remain capable of renewed division upon stimulation. In both single-celled and multicellular organisms, quiescence plays a crucial role in maintaining cellular integrity, tissue homeostasis, and overall organismal health [1]. 

In mammals, quiescence also plays a crucial role in regulating the balance between cell proliferation and differentiation [2]. For example, during neural development, neuroblast cells enter a state of developmental quiescence, which temporarily stops cell division and ensures proper differentiation. Similarly, adaptive quiescence, such as that observed in T cells, enables the immune system to maintain a pool of non-activated, yet responsive, T cells that can quickly proliferate upon encountering a specific antigen [2]. Stem cells also remain in a quiescent state until they are triggered to proliferate, either to replace damaged cells or due to an oncogenic change, which can lead to the development of cancer [1]. 

For unicellular microbes, quiescence is the most common state in nature and it is crucial for long-term survival under the prevailing unfavorable conditions. Indeed, proliferation may be a relatively rare event in the life history of a microbe, as the nutrients that provide the energy necessary for biomass accumulation and cell division are only temporarily available in the environment. Although the definition of quiescence, namely that quiescence is a temporary and reversible growth-arrested state that cells can enter in response to unfavorable environmental conditions [3], captures the essence of the quiescent state, it is difficult to set the boundaries of when and under what circumstances a given cell or population of cells is quiescent.

This systematic review encompasses research conducted specifically on the budding yeast *Saccharomyces cerevisiae*, an excellent eukaryotic model organism frequently used in a variety of fields within the natural sciences [4]. Importantly, fundamental biological processes are conserved among eukaryotes, and the cellular organization of the yeast cell is similar to that observed in higher organisms, including humans [5]. Many fundamental biological processes have been described for the first time in budding yeast, including recent work on the mechanisms of apoptosis, for which the Nobel Prize was awarded in 2016 [6]. Because *S. cerevisiae* can be used for such a broad range of research, we believe that this systematic review provides a good overview of the current state of quiescence research.

Although the terms “quiescent”, “stationary phase” and “G0” are often used interchangeably [7], we suggest separating them, following the proposition formulated almost two decades ago [8]. This proposition is that the “stationary phase” is a part of the microbial culture growth curve, which indicates that a given population has used all available its resources and has reached maximum density for a given condition. It is important to stress that not all non-proliferating cells present in the stationary phase are quiescent. Senescent cells are unable to resume cell division and will die in the future. Additionally, in the stationary-phase population, there are cells of varying age, as well as the dead ones; therefore, such a population comprises cells in diverse physiological states rather than quiescent cells only. “G0” refers to a cell that has exited the cell cycle, typically from the G1 phase, and it does not indicate whether this arrest is reversible. The term “quiescent” describes cells’ ability to survive and their capacity to re-enter the cell cycle. The other form of growth arrest can be observed in diploid yeast strains that can sporulate via meiosis. Although yeast spores and quiescent cells share common characteristics, with the most important being their ability to resume growth, their underlying molecular mechanisms are different [9]; therefore, research articles studying spores are excluded from this review.

Heterogeneity within stationary-phase yeast populations was first described in 2006. The classic publication by Margaret Werner-Washburne’s group [10] described a method via which to isolate two subpopulations from the stationary phase using density centrifugation: the less dense upper fraction, called non-quiescent (NQ), and the denser lower fraction, called quiescent (Q). In the same year, Yang et al. (2006) [11] identified five phenotypes in the stationary phase based on nuclei morphology and the degree of chromatin condensation.

In a laboratory set-up, quiescence is usually triggered by gradual carbon starvation; however, cells can enter the quiescent state in response to the limitation of other key nutrients too. Importantly, depending on the limiting nutrient, the cellular response may differ [12]. There are distinct genes that are needed for a cell to survive the starvation of a particular nutrient and the resulting metabolic profiles of starved cells are also different [12]. For example, respiration and functional mitochondria are crucial for survival during glucose starvation, while vacuole and autophagic pathways are needed in nitrogen starvation [12,13]. Additionally, the ability of cells to synthesize all compounds required for growth (prototrophy) is crucial for surviving starvation [14,15]. Recently, Santos et al. (2021) [15] conducted a high-throughput analysis and concluded that due to the complex interplay between the strain genetic background (auxo/prototrophy), gene deletions, and media composition, quiescence should be studied using prototrophic strains only. Taken together, there is no one universal quiescent state, but rather a combination of environmental effects and cellular variations that lead to reversible growth arrest [16,17]. 

Growth arrest is associated with a variety of cellular rearrangements; however, the presence or lack of those characteristics is not enough to predict cell fate, particularly whether a given cell will or will not be able to resume growth under favorable conditions. Importantly, entry into quiescence is a gradual process that can take days to establish and it is not common to all cells. The actin skeleton is transformed into spheroid actin bodies, the mitochondrial network is condensed into multiple vesicles, and there is an accumulation of storage materials, particularly trehalose [16,17,18,19]. Not only are organelles rearranged, but the genome is also adjusted, including global chromosome condensation and telomere clustering; meanwhile, proteins, enzymes, and mRNAs are condensed and encapsulated into stress granules and P-bodies [17,20,21,22]. Note, however, that not only can these characteristics vary from cell to cell (i.e., not all Q cells will share the same universal characteristic), but they can also change over time [17]. In particular, the longer a cell remains in the quiescent state, the more time it may need to re-enter the cell cycle [23]. In addition, after a long period of time, quiescent cells can accumulate damages that commit them to senescence and subsequently to apoptosis [17]. 

Quiescence provides protection from a variety of environmental stressors. For example, quiescent cells have been shown to be more resistant to high temperatures [18], oxidative stress [12], antibiotic treatment [24], and prolonged starvation [25]. 

The essence of the quiescent state, namely the ability to re-enter the cell cycle, makes the study of quiescent cells extremely challenging. This is because some assumptions and/or predictions about the fate of non-proliferating cells have to be made before such a cell can prove its ability to resume growth. Unfortunately, given the diversity of scenarios, it seems impossible to provide a more precise and rigorous definition of quiescence.

The aim of a systematic review is to provide a comprehensive and unbiased synthesis of findings in the area of interest. We use the systematic review methodology to capture and categorize the current state and diversity of all published research conducted on *S. cerevisiae* quiescent cells. This involves searching both scientific databases and classified article texts using pre-planned procedures (see Section 2 for details), which minimalizes the bias and ensures that all relevant articles are taken into account. A systematic review focuses on summarizing and synthesizing research, while a meta-analysis, which may follow, goes a step further by quantitatively analyzing the effect sizes across studies. An analysis of effect sizes is not performed in this review. Following the recommendations of Foo et al. (2021) [26], we identified the objectives of the review (described below), then formulated and tested search strings. We then performed an initial screening, which is a classification of research articles based on the following: *titles*, *abstracts*, and *keywords*. Finally, we conducted a full-text screening of the selected articles (see details in the Section 2). 

We classified the selected articles according to the following information: (1) what the authors mean by “quiescence”, i.e., whether the whole stationary-phase population is treated as quiescent, or some subpopulations are distinguished within the stationary-phase culture; (2) how old the studied populations/cells are; and (3) how quiescence is induced. We also noted (4) the metabolic profile of a strain, in particular whether it is prototrophic or auxotrophic, and its origin (laboratory or wild). We further recorded which (5) biological aspects are studied in the analyzed research article in order to illustrate how broad the implications of quiescence can be. Moreover, we analyzed how these features change over the years. We combined the above features and identified the most and the least frequent experimental set-ups. Finally, we propose a set of guidelines that, we believe, can improve research clarity and facilitate knowledge transfer. We also discuss several directions that the field of quiescent could pursue.

## 2. Methods

### 2.1. Literature Search

**Figure 1:**
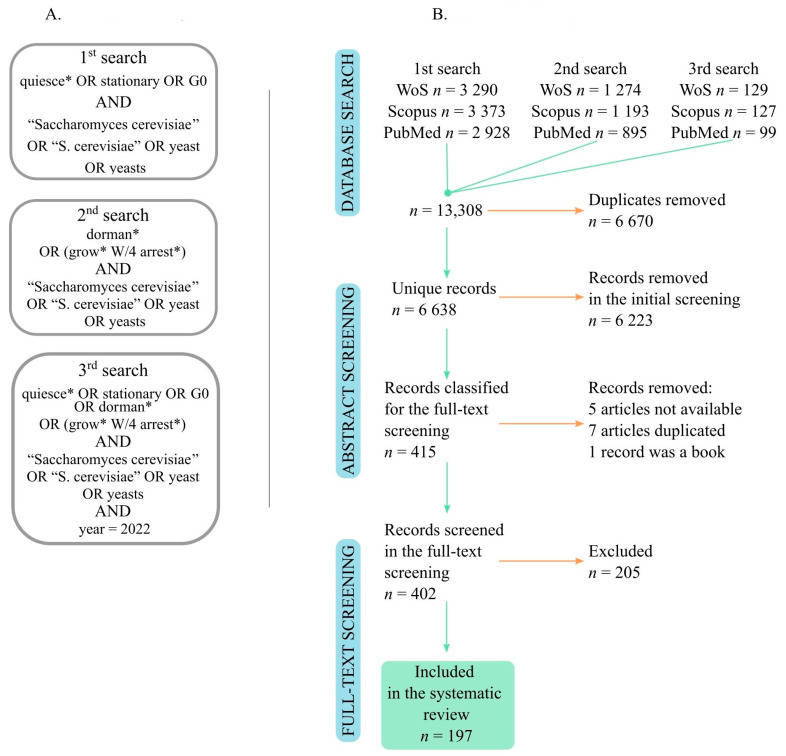
(**A**) The search strings used to find records in the databases. The asterisk (*) indicates a wildcard character for any combination of characters that could follow the prefix. See the Supplementary Materials for details. (**B**) The PRISMA diagram of the classification of records in this systematic review. See the Supplementary Materials for details.

The systematic review was performed following the Preferred Reporting Items for Systematic Reviews and Meta-Analyses (PRISMA) guidelines [27]. We searched three databases (Scopus, Web of Science and PubMed) for records published up to the end of 2022 (Figure 1A, see search strings in the Supplementary Materials). The deduplicated records were uploaded to Rayyan (https://www.rayyan.ai/ accessed on 1 December 2021) for the initial screening based on titles, abstracts and keywords. The screening was carried out independently by three scientists. The records were classified according to the decision tree (Supplementary Materials). Specifically, we excluded articles that were not written in English, those that did not use *S. cerevisiae*, and all publications types other than original research articles. We excluded articles in which “stationary phase” was not the subject of the research (e.g., the stationary phase was used only to refer to the age of a population). The full texts of the 402 included records were then acquired using Zotero reference manager (https://www.zotero.org/ accessed on 1 December 2021). Three scientists performed the full-text analysis, each analyzing ~130 articles. At this stage, 205 articles were excluded, and the reasons for their exclusion are provided in the Supplementary File. Finally, 197 articles were included and analyzed within this systematic review. The database and literature search procedures are summarized in a PRISMA diagram (Figure 1A,B). The selected articles were screened for the following features: (1) meaning of quiescence, (2) age of studied populations, (3) method used to induce quiescence, (4) metabolic profile and origin of used strains, and (5) biological aspects studied within the given article (see details in Tables S1–S5). In all cases (except for metabolic profile), the categories distinguished within a feature were non-exclusive, i.e., a single research article could be classified into more than one category within a single feature. 

### 2.2. Data Analysis and Visualisation

The data analysis and visualizations were conducted in R 4.1.2 [28] using the following packages: dplyr [29], readxl [30], ggplot2 [31] and easyalluvial [32]. Schemes were prepared in Inkscape [33].

The word cloud figure was generated using the R package wordcloud2 [34]. The combined text of the title and the abstract of all 197 articles included in this review were used. The word cloud was restricted to words occurring 20 or more times, and the biologically irrelevant words were manually removed. This left 133 words to generate the word cloud. 

## 3. Results

### 3.1. Literature Search

The Web of Science, Scopus and PubMed databases were searched three times. The first search was performed using the keywords *quiescence*, *stationary* and G0. However, later, we decided to extend the search for additional keywords: *dormancy* and *growth arrest*. The third search was performed to update the records for the research articles published until the end of 2022 (Figure 1, see the details in the Supplementary Materials).

**Figure 2:**
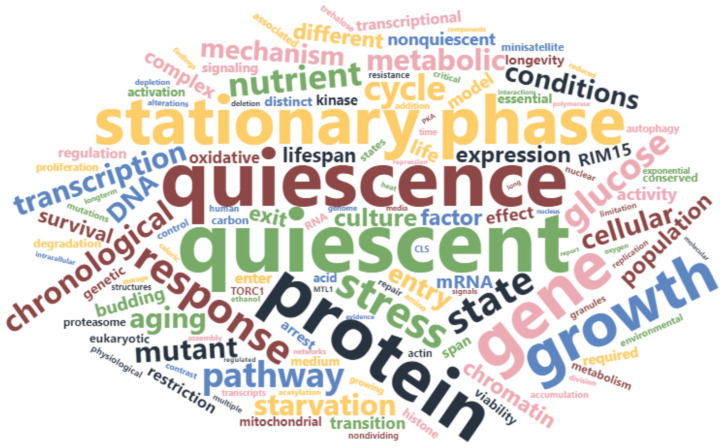
The word cloud of the most frequently used terms in the titles and abstracts of the research articles included in this systematic review. This word cloud serves to highlight the terms that are most frequently associated with the quiescent state, providing valuable insights into the prevailing research trends and themes within the field.

More than 6.5 thousand unique records were uploaded for initial screening based on their titles, abstracts, and keywords, out of which 402 were classified for full-text screening. Finally, 197 articles were selected for further analysis (Figure 1B). The original research articles included in this systematic review are as follows: [10,11,12,13,15,18,19,20,21,22,23,25,35,36,37,38,39,40,41,42,43,44,45,46,47,48,49,50,51,52,53,54,55,56,57,58,59,60,61,62,63,64,65,66,67,68,69,70,71,72,73,74,75,76,77,78,79,80,81,82,83,84,85,86,87,88,89,90,91,92,93,94,95,96,97,98,99,100,101,102,103,104,105,106,107,108,109,110,111,112,113,114,115,116,117,118,119,120,121,122,123,124,125,126,127,128,129,130,131,132,133,134,135,136,137,138,139,140,141,142,143,144,145,146,147,148,149,150,151,152,153,154,155,156,157,158,159,160,161,162,163,164,165,166,167,168,169,170,171,172,173,174,175,176,177,178,179,180,181,182,183,184,185,186,187,188,189,190,191,192,193,194,195,196,197,198,199,200,201,202,203,204,205,206,207,208,209,210,211,212,213,214,215,216,217,218]. The most frequently used terms in the titles and abstracts of the included research articles are represented in the word cloud (Figure 2). 

### 3.2. The Meaning of “Quiescence” 

**Figure 3:**
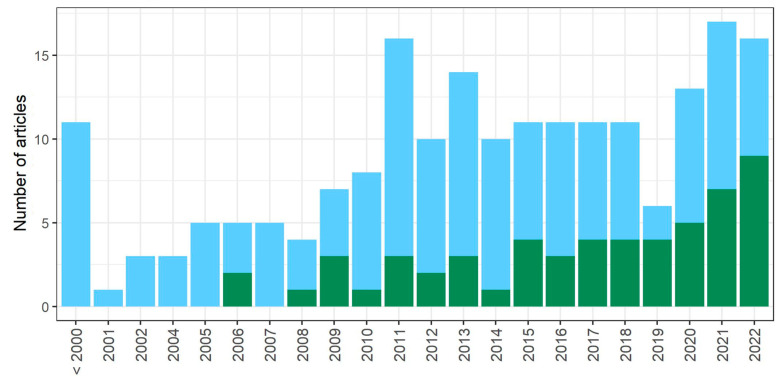
An overview of the approaches used to define quiescent populations over the years. Blue corresponds to articles in which the whole stationary-phase population (*whole_pop*) is treated as quiescent, while green corresponds to articles in which some subpopulations are distinguished within the stationary-phase culture (*subpop*).

We analyzed whether, within a given publication, the whole stationary-phase population is treated as quiescent (*whole_pop*), or whether some phenotypically distinct subpopulations are distinguished (*subpop)*, (Figure 3, Table S1). Research on non-purified whole stationary-phase cultures prevails; however, in recent years, authors have more frequently considered the phenotypic variability of cells within the stationary-phase population. Among all the articles analyzed, 28% take into account the heterogeneity of SP, while from 2015, this percentage increases to 42%, and in the last 3 years (from 2020), subpopulations are distinguished in 46% of the articles. Out of the research articles that consider SP heterogeneity, 86% use the density gradient fractionation procedure proposed by the Werner-Washburne group [10]. 

### 3.3. The Age of Studied Quiescent Populations or Cells 

**Figure 4:**
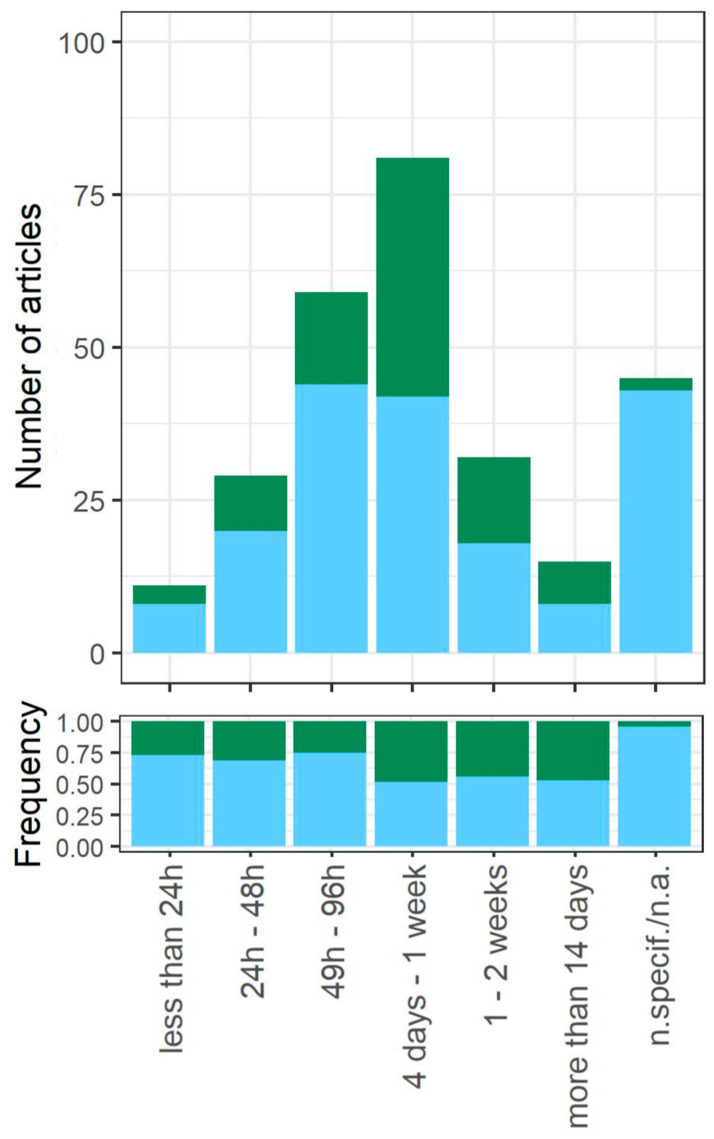
The distribution of the age of studied quiescent populations among the analyzed research articles, shown as an absolute number of publications and frequencies. Blue corresponds to articles in which the whole stationary-phase population (*whole_pop*) is treated as quiescent, while green corresponds to articles in which some subpopulations are distinguished within the stationary-phase culture (*subpop*).

Within this review, we classify the chronological age of the population into 6 categories (Table S2). The majority of research focusing on quiescence is carried out on 2–7-day-old populations (61% of articles) (Figure 4). Interestingly, in older populations (age categories more than 4 days of growth), a similar number of articles treat the whole SP population as quiescent and distinguish subpopulations in the SP. Additionally, we could not assign the age of the population in 45 articles. These include studies in which the age was not clearly specified and theoretical (modelling) studies that were based on already existing datasets.

### 3.4. Starvation Induction

**Figure 5:**
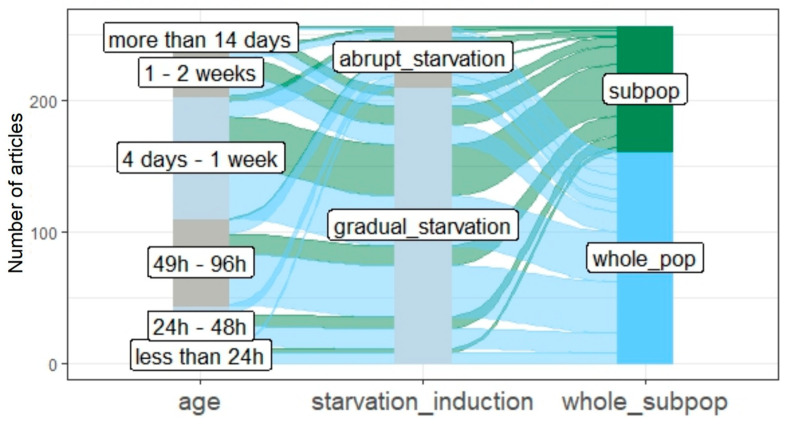
The visualization of dependencies between features of studied quiescent populations. The first column (*age*) corresponds to the categories within the feature *age of studied populations or cells* (Figure 4). The second column (*starv_induction*) corresponds to the method by which starvation was induced, the third column (*whole*_*subpop*), as well as the colors, correspond to the meaning of quiescence adopted within a given research article.

In most research articles, to obtain quiescent cells, populations were kept in a growth medium for a certain period of time (chronological age, *gradual_starvation*, 86% of articles, Figure 5, Table S3). However, we also distinguished articles in which cells were arrested by transfer from rich to starvation medium or by the addition of growth-arresting factors (e.g., rapamycin or alpha-factor, referred to as *abrupt*_*starvation*, in 24% of articles). We did not recognize any pronounced patterns or interdependences between the populations’ age, the method of starvation induction, and the definition of quiescence applied in a given research set-up (Figure 5).

### 3.5. The Metabolic Profile and Origin of Studied Strains 

**Figure 6:**
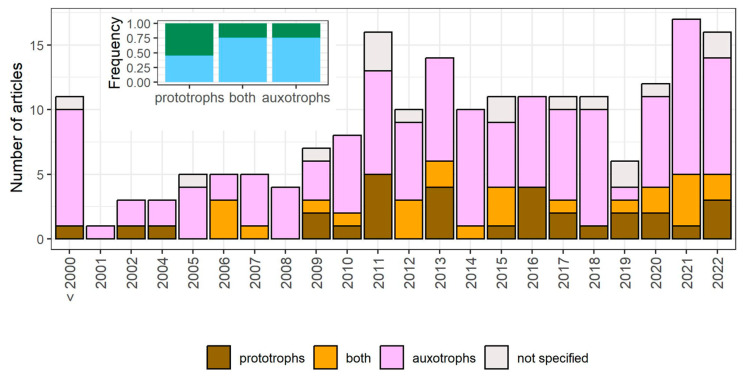
Usage of *prototrophic* (brown), *auxotrophic* (purple) and both prototrophic and auxotrophic (orange) strains of *S. cerevisiae* over time and the total frequency of each metabolic profile. On the frequency plot, blue corresponds to articles in which the whole stationary-phase population (*whole_pop*) is treated as quiescent, while green corresponds to articles in which some subpopulations are distinguished within the stationary-phase culture (*subpop*).

We report whether *prototrophic* or *auxotrophic* strains were used within a given research article (Figure 6, Table S4). The majority of research has been carried out using auxotrophic strains only (63% of articles). The use of prototrophic strains was rare before the 2010s (22%) and between 2020–2023, it increased to 31% (Figure 6). Interestingly, research conducted using prototrophic strains more frequently distinguishes the subpopulations in the stationary-phase cultures (χ^2^ = 11.97, df = 2, *p* = 0.0025). We also noted whether the origin of the strain was *lab* (laboratory) or *non-lab* (including wild and industrial strains). Currently, research on quiescence seems to be limited to laboratory strains. We recognized only five research articles in which *non-laboratory* strains were studied, all of which were fermentative strains used in the production of alcoholic beverages. 

### 3.6. Biological Aspects

**Figure 7:**
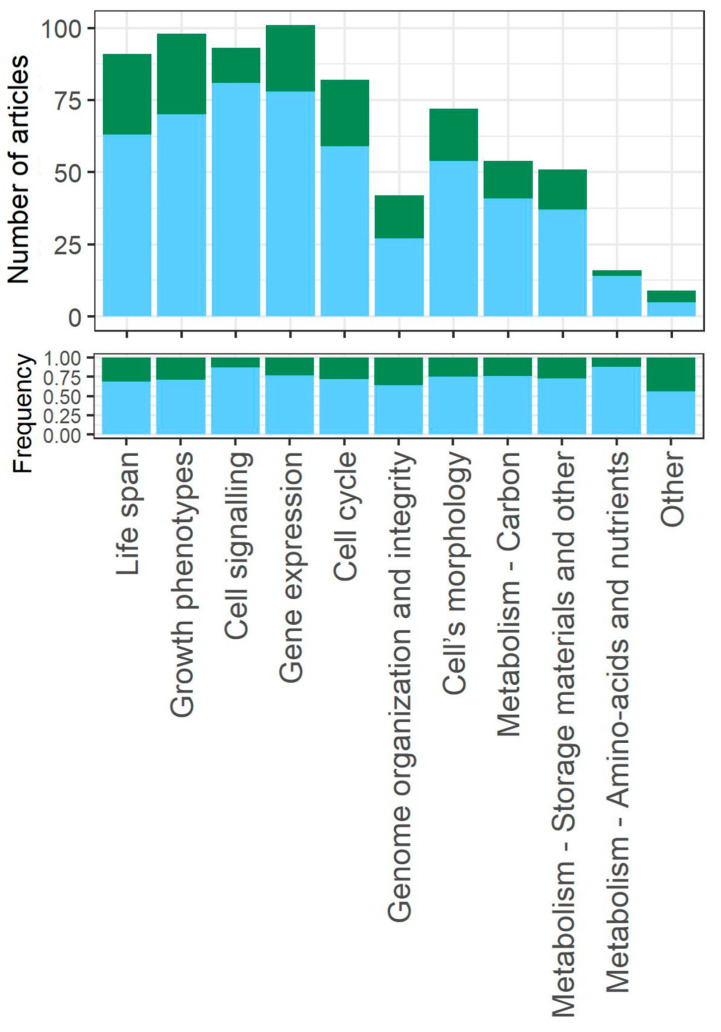
Number of articles and frequency of the biological aspects studied within the research articles included in this systematic review. Blue corresponds to articles in which the whole stationary-phase population (*whole_pop*) is treated as quiescent, while green corresponds to articles in which some subpopulations are distinguished within the stationary-phase culture (*subpop*).

We identified 10 categories of biological aspects that were studied in the analyzed research articles (Figure 7). Details of all the categories can be found in the Supplementary Materials (Table S6). Note that the categories are not exclusive, i.e., several biological aspects can be studied within a single research article. 

Within all 197 research articles analyzed, the most frequently studied category was *gene expression* (100 articles), which includes RNA-based investigations as well as the mechanism and regulation of protein synthesis. The transcriptomic profiles of proliferating and quiescent cells differ significantly. Moreover, transcription in quiescent cells can be further influenced by various factors, such as environmental signals and the genetic background. Consequently, the analysis of gene expression patterns appears to be a significant aspect of numerous research articles. The *cell signalling* category (93 articles), which gathers research on various signalling pathways, was also very common. There are two ecological categories: *life span* (91 articles), which focuses on aspects related to chronological ageing, and *growth phenotypes* (98 articles), which includes research articles that have studied various population features, such as cells’ survival rate or their ability to grow in response to stressors. Quiescence entry in the context of the *cell cycle* was studied in 82 articles, and *genome stability* was the least frequent aspect, studied in 41 articles. Microscopic investigations of *cell morphology* were conducted by the authors of 72 articles. Studies related to *metabolism* (altogether 83 articles) fall into three categories: those related to *carbon metabolism* (54 articles), *amino acids and nutrients* (16 articles), and *storage materials* (51 articles). 

### 3.7. Experimental Set-Up 

**Figure 8:**
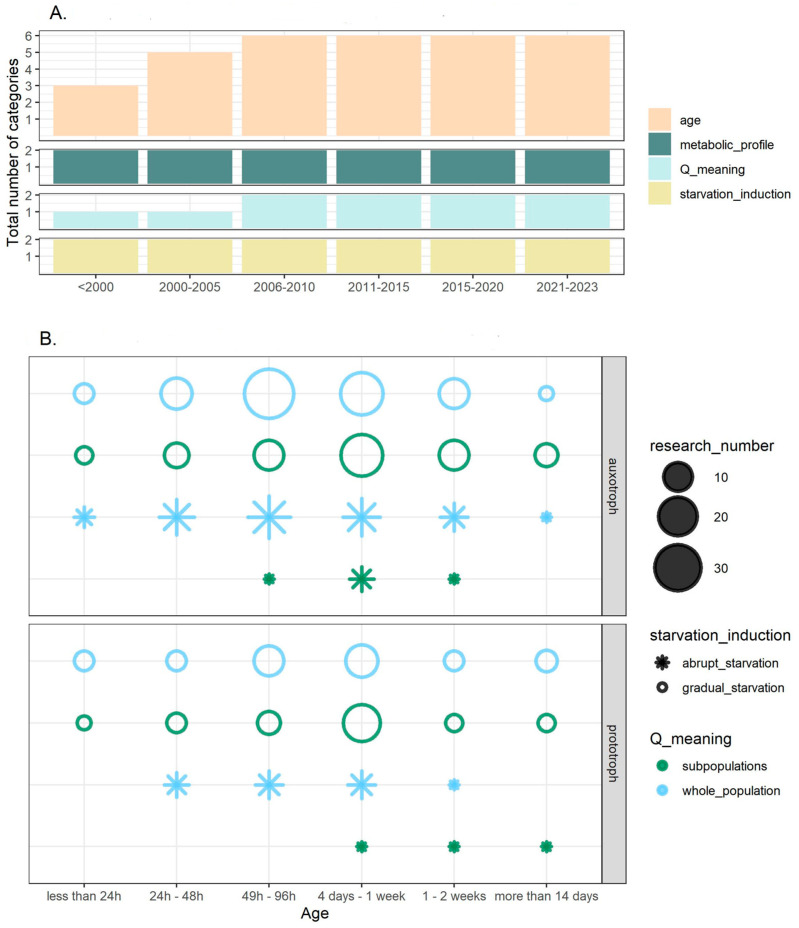
Visualization of experimental set-ups used to study quiescence. (**A**) Number of categories (*y*-axis) within a given feature (panels and colors) present in the research articles in the given time frame. The maximal y-value in a given panel corresponds to the total number of categories within the feature; for example, we distinguish two categories within the feature *Q_meaning* (*whole_pop* and *subpop*) and six categories within the *age* feature (*less than 24 h*, *24 h–48 h*, *49–96 h*, *4 d–1 w*, *1–2 w*, *more than 14 d*). (**B**) Visualization of frequencies of experimental set-ups. The size of points corresponds to the number of articles within the given experimental set-up; if the point is missing, it means that no research was conducted using that experimental set-up. The shape of the point (circle/star) corresponds to the way in which quiescence was *induced* and the color corresponds to *the meaning of quiescence*. The *metabolic profile* is divided into two panels and *age* is plotted on the *x*-axis.

Using the categorization proposed within this review, the number of possible experimental set-ups could be as high as 48 (*age* (6 categories) × *metabolic profile* (2 categories) × *quiescence meaning* (2 categories) × *starvation induction* (2 categories)) (Figure 8). We identified the most frequent combination, which is an auxotrophic population gradually starved for 49–96 h (2–3 days) and analyzed as a whole population (without distinguishing subpopulations). This combination occurred in 35 research articles. Two other frequent combinations involve a auxotrophic population gradually starved for 4–7 days and analyzed as a whole population (25 articles) or with the identification of subpopulations (24 articles). The most frequent experimental set-up with prototrophic strain(s) occurred 18 times, in which populations were gradually starved for 4–7 days and analyzed with the identification of subpopulations. Eight combinations do not occur in any article. 

## 4. Discussion

This systematic review presents the diversity of research on the quiescent state in the eukaryotic cell model organism of the yeast *Saccharomyces cerevisiae*. Quiescence is a broad term; therefore, to ensure the transparency and reproducibility of research across different laboratories and to facilitate knowledge transfer, it is important to define the crucial variables that vary between the research.

The major division within the research on quiescence is what the authors consider quiescence to be, namely whether the whole starved/stationary-phase population is treated as quiescent, or whether some phenotypic heterogeneity is acknowledged via the identification of distinct subpopulations within the stationary-phase population. The technique most widely used to separate quiescent (lower fraction) cells was invented in 2006 in the laboratory of Margaret Werner-Washburne [10]. The fractionation procedure is based on the assumption that growth-arrested cells tend to be denser due to the accumulation of storage materials, and as such, they can be separated via centrifugation on a density gradient. However, both the biological state and nomenclature of the subpopulations separated via the fractionation procedure are confusing. While some researchers have adopted the terms “Quiescent and Non-Quiescent”, others prefer to name them “Upper and Lower fractions” (both nomenclatures used in the original work [10]). Their argument is that all cells in the stationary-phase population that restart division when nutrients are available are Q, including some cells called NQ, and that the above method rather separates cells with a low and high amount of storage material [19]. Other identification methods use hallmarks such as histone methylation landscapes [71], the mitochondrial network morphology [19], or cytoplasmic granules [18]. However, these methods only enable the identification of heterogeneity, and not the physical separation of subpopulations and independent testing. Indeed, given the natural heterogeneity of stationary-phase populations, any attempt to physically separate subpopulations may be considered overly simplistic. We believe that this is the most striking gap in knowledge and technology. Namely, we recognize the great need to connect population-based research (mostly on Q and NQ subpopulations) with the cellular heterogeneity recognized by studies on single cells. 

Although the presence of phenotypic heterogeneity in stationary-phase populations is well documented, the extent to which this heterogeneity affects population characteristics, as well as adaptations to specific conditions, is frequently unknown. For example, after experimental evolution aimed at enriching the population for quiescent or non-quiescent cells (Lower and Upper fractions, respectively), the proportion of Q (L) cells varied from 95% (Q-enriched) to 13% (NQ-enriched), whereas the ancestral strain had 75% Q cells [67]. In this case, the Q-enriched population can be roughly treated as homogeneous, while making the same assumption for the NQ-enriched population would be incorrect. On the other hand, the article by Sagot and Laporte (2019b) [17] shows heterogeneity even within quiescent cells, which are often believed to fall mostly into the Lower fraction. Altogether, the heterogeneity of the stationary-phase population needs to be taken into account in order to make further assumptions, although physical separation into multiple homogeneous subpopulations may be impossible or may additionally affect cells’ entry into quiescence and/or cellular properties. 

Cell properties change with the time spent in starvation, during the so-called chronological ageing [7,17]. The transition to quiescence begins before nutrients are exhausted, and the gradual slowing down of metabolic activity takes days to establish (see review [7] for details). Authors should therefore carefully decide upon the time point at which they determine and name the cell or population to be “quiescent”. In particular, they should decide how long the population should be kept in starvation for all cells to achieve the quiescent state and whether, for example, 2-day-old and 7-day-old quiescent cells are significantly different, taking into account the specific conditions of a given experiment. The commonly used statement declaring that “the population has been grown to stationary phase” traditionally refers to a 24 h old, overnight, population with an average density of 2 × 10^8^ cell/mL. However, this description is not precise enough for quiescence identification. The physiological state of the cell changes with age, particularly at the onset of starvation (approximately up to 7 days), so chronological age is an important feature of the population. In 45 articles, we failed to assign the correct age to the populations being studied. This might cause major problems in the interpretation of results.

Another aspect related to nutrient limitation is the method applied in order to induce starvation/quiescence entry. We noted whether cells were allowed to adapt slowly to the decreasing amount of nutrients, or whether they were suddenly transferred to starvation media and/or a growth-arresting treatment was applied. This classification is important because gradual nutrient limitation leads to a different physiological state to that induced by an abrupt transfer to a nutrient-limited environment. In particular, nutrient transporters have varying levels of efficiency, which are adapted to the availability of a given substance in the environment. Consequently, the gradual depletion of a nutrient is reflected in progressive switches in the appropriate transporters, which is not the case when the environment is suddenly changed [7]. Although cells are adapted to gradually decreasing nutrients, both *abrupt* and *gradual* quiescence inductions can mimic naturally occurring ecological scenarios. For example, abrupt nutrient deprivation can occur when yeasts growing on ripening fruit are suddenly washed into the soil by rain. Nevertheless, the way in which quiescence entry is induced is a major signal and can influence a variety of cell properties. Therefore, we argue that the way in which starvation is induced should be thoroughly described in the article, and not just mentioned in the Section 2, especially in the case of the less frequently used methods of abrupt growth arrest.

Quiescence entry is a complicated and multi-step process that requires energy and extensive adjustments in cell functioning. Thus, any genetic disruption, especially one that affects nutrient uptake and amino acid synthesis, has a pronounced effect on cellular properties and consequently on survival [14]. Within this review, we report whether *prototrophic* or *auxotrophic* (or both) strains were used. There is evidence that auxotrophs may be short-lived and lack the characteristic of quiescence, due to disruptions in growth-controlling pathways [14,15]. Moreover, amino acid over-supplementation in order to compensate for genetic defects causes further nutritional imbalances that additionally shorten the chronological life span [7,14]. Laboratory strains with trophic markers (auxotrophs) are widely used because they are easy to select on a specific media. However, gene deletions, such as those seen in auxotrophs, have a pronounced effect on cell function and may interfere with proper quiescence entry. Furthermore, when using auxotrophic strains in research, it may be difficult to distinguish the effect being studied from the effect of disrupted amino acid synthesis. For example, it has been shown that quiescent cells are more sensitive to UV radiation [105], but as this was carried out using auxotrophic strains, it is unclear whether the same sensitivity would be seen when testing prototrophs. According to current knowledge, auxotrophs should be avoided when studying quiescence [7]. 

We were also interested in whether research on *S. cerevisiae* quiescence is limited to laboratory strains or whether there have been some attempts to study this phenomenon in wild/non-laboratory strains. We recognized only five research articles in which *non-laboratory* strains were used [47,58,72,87,191]. Laboratory strains are unlikely to be good representatives of microbes isolated from nature [219]. There is a growing interest in wild yeast [220], so studying quiescence in wild isolates can provide valuable insights, for example, into how universal traits are, even just within the *Saccharomyces* sp. group. 

The fundamental importance of quiescence is reflected in the broad range of research fields (biological aspects) we identified (Figure 7). Quiescence can be studied at the gene-level in order to discover the mechanisms responsible for the transition, the biochemical processes that enable this cellular response, and finally how these changes affect ecological aspects, such as cells’ survival and resistance to environmental changes. 

Finally, we investigated the experimental setups used to study quiescence by combining the information on the populations under study. We report that since 2006, all the categories of analyzed features that we defined are present in the experimental set-ups (Figure 8A), which means that the diversity of approaches used to study quiescent state is steadily present in research. We also analyzed which experimental set-ups are the most and the least frequently used to study quiescence, which can help to identify knowledge gaps. In particular, there are eight theoretically possible experimental set-ups that have never been used to study quiescence in budding yeasts (Figure 8B). 

We also noticed an interesting connection between the age of the studied population and the frequency of the subpopulations being distinguished within the SP (Figure 4). In particular, the older the population studied, the more frequently subpopulations are distinguished. Although this correlation is not statistically significant (Spearman rank correlation r = 0.65, *p* = 0.17), we recognize this as an interesting research area. Cells tend to be more homogeneous in younger populations; in particular, cells can synchronize during proliferation, and the diversification starts when nutrients become limiting. The heterogeneity increases with the time spent in starvation, as the fraction of cells that enter quiescence can increase, while older/damaged cells commit to senescence or apoptosis. Although in bacteria survival during the stationary phase (LTSP) and the evolution of growth advantage in stationary-phase (GASP) mutants are well studied [221,222], there are only limited reports regarding yeast [223]. Indeed, studies on the evolution of quiescence and phenotypic heterogeneity in the long-standing stationary phase are potential intriguing future research directions.

Our systematic review could be a valuable source of information for higher-level comparisons, such as a meta-analysis. The database we have compiled makes it easy to identify studies that have been conducted using a specific methodology and contain empirical results. This tool can be used to address a variety of research questions, such as the effect of metabolic profile on quiescent characteristics. 

In conclusion, this review demonstrates how diverse the understanding of quiescence can be within research articles on *Saccharomyces cerevisiae*. Based on this review, we have prepared guidelines for publishing authors (Box 1). It is crucial to report the following: whether the whole population was treated as quiescent, or some subpopulations were distinguished; what the chronological age of the quiescent populations/cells studied was; how quiescence was induced (i.e., whether the population was left to starve in growth media or an abrupt environmental change occurred); and whether the strain(s) used were prototrophic or auxotrophic. These features should not only be carefully reported in the Section 2, but should also be considered when designing an experiment. We encourage the scientific community to provide more detailed justification and explanation for their research. We believe that such details will facilitate knowledge transfer and stimulate valuable scientific debate.

Box 1 **Guidelines on what information should be provided by authors when studying quiescence in the budding yeast.**

**Guidelines for the research on yeast quiescence**
1. Define studied quiescent cells
◦Do you treat the whole stationary-phase population as a homogenous entity with only quiescent cells, or do you distinguish cell subpopulations with distinct phenotypes?◦What method do you use to separate/identify cell subpopulations/phenotypic heterogeneity? Is this method described in the literature or is it novel? If possible, describe how the used methodology relates to other methods of identifying phenotypic heterogeneity in the stationary-phase populations.
2. Specify how quiescence is induced
and the time when you assume the population entered quiescence
◦How is quiescence induced? Do you leave cultures to starve for some time (how long?) and allow cells to adjust to gradual nutrient depletion? Do you use any growth-arresting reagents? How long is a population left to grow before growth-arresting treatment is applied?◦How long does the population grow before the conduction of tests/experiments?◦What is the growth-limiting nutrient? ◦Specify the growth conditions (e.g., initial cell density, inoculum size, media composition and volume, inoculum size).
3. DEFINE THE METABOLIC PROFILE AND GENETIC BACKGROUND OF USED STRAINS
◦Is the strain auxotrophic or prototrophic? Specify all trophic markers, and if possible, describe how such auxotrophies influence the quiescent state.◦Specify strain name and genotype (other than trophic markers).

## 5. Conclusions

We used a systematic review approach to comprehensively analyze the main methodological aspects of studying quiescence in *Saccharomyces cerevisiae*. Our review shows that the understanding of quiescence in this model organism is very diverse, reflects researchers’ scientific perspective, and reflects the questions asked. We have observed trends in how the perception of quiescence has evolved over time. For instance, subpopulations are increasingly being recognized, and there is a growing interest in prototrophic strains. Importantly, there are no biases in the methodological approaches that have been used in studies of the different biological aspects. On the one hand, our work highlights the lack of standardization in reporting the experimental details of quiescent cells and populations, which can be confusing and misleading when drawing general conclusions. On the other hand, the diversity of methodological approaches used in the published studies may be useful for higher-level comparisons that could be made using meta-analyses. Our catalogue of studies in each category could be very useful for this purpose.

Although we have identified the most frequent experimental set-ups, it is important to note that within the research community, there is no single dominant protocol for quiescence research. To study yeast quiescent cells that are a fraction of the stationary-phase population, we recommend using prototrophic strains, gradually starved for at least 4 days. However, we believe that given the current state of knowledge, it is impossible to provide strict recommendations regarding the best approach to studying quiescence. Therefore, we focus on highlighting the crucial features of the quiescent population under study (Box 1) and encourage researchers to consider the benefits and constraints of various approaches. 

## Data Availability

The data presented in this study are available in the Supplementary Materials.

## Supplementary Materials

The supporting information can be downloaded at: https://www.mdpi.com/article/10.3390/cells12121608/s1. In the Supplementary File there are the search strings, the decision tree, and Tables S1–S5 with the details on the categorization used during full-text screening. In addition we share the excel file with all analyzed information on research articles included in this systematic review.

## References

[1] O’Farrell P.H. (2011). Quiescence: Early evolutionary origins and universality do not imply uniformity. Philos. Trans. R. Soc. B Biol. Sci..

[2] Marescal O., Cheeseman I.M. (2020). Cellular Mechanisms and Regulation of Quiescence. Dev. Cell.

[3] De Virgilio C. (2012). The essence of yeast quiescence. FEMS Microbiol. Rev..

[4] Karathia H., Vilaprinyo E., Sorribas A., Alves R. (2011). *Saccharomyces cerevisiae* as a model organism: A comparative study. PLoS ONE.

[5] Barnett J.A. (2007). A history of research on yeasts 10: Foundations of yeast genetics1. Yeast.

[6] Hohmann S. (2016). Nobel yeast research. FEMS Yeast Res..

[7] Breeden L.L., Tsukiyama T. (2022). Quiescence in *Saccharomyces cerevisiae*. Annu. Rev. Genet..

[8] Gray J.V., Petsko G.A., Johnston G.C., Ringe D., Singer R.A., Werner-Washburne M. (2004). “Sleeping Beauty”: Quiescence in *Saccharomyces cerevisiae*. Microbiol. Mol. Biol. Rev..

[9] Honigberg S.M. (2016). Similar environments but diverse fates: Responses of budding yeast to nutrient deprivation. Microb. Cell.

[10] Allen C., Büttner S., Aragon A.D., Thomas J.A., Meirelles O., Jaetao J.E., Benn D., Ruby S.W., Veenhuis M., Madeo F. (2006). Isolation of quiescent and nonquiescent cells from yeast stationary-phase cultures. J. Cell Biol..

[11] Yang H., Ren Q., Zhang Z. (2006). Chromosome or chromatin condensation leads to meiosis or apoptosis in stationary yeast (*Saccharomyces cerevisiae*) cells. FEMS Yeast Res..

[12] Klosinska M.M., Crutchfield C.A., Bradley P.H., Rabinowitz J.D., Broach J.R. (2011). Yeast cells can access distinct quiescent states. Genes Dev..

[13] Jacquel B., Aspert T., Laporte D., Sagot I., Charvin G. (2021). Monitoring single-cell dynamics of entry into quiescence during an unperturbed life cycle. eLife.

[14] Boer V.M., Amini S., Botstein D. (2008). Influence of genotype and nutrition on survival and metabolism of starving yeast. Proc. Natl. Acad. Sci. USA.

[15] Santos S.M., Laflin S., Broadway A., Burnet C., Hartheimer J., Rodgers J., Smith D.L., Hartman J.L. (2021). High-resolution yeast quiescence profiling in human-like media reveals complex influences of auxotrophy and nutrient availability. GeroScience.

[16] Sagot I., Laporte D. (2019). Quiescence, an individual journey. Curr. Genet..

[17] Sagot I., Laporte D. (2019). The cell biology of quiescent yeast—A diversity of individual scenarios. J. Cell Sci..

[18] Lee H.-Y.Y., Cheng K.-Y.Y., Chao J.-C.C., Leu J.-Y.Y. (2016). Differentiated cytoplasmic granule formation in quiescent and non-quiescent cells upon chronological aging. Microb. Cell.

[19] Laporte D., Gouleme L., Jimenez L., Khemiri I., Sagot I. (2018). Mitochondria reorganization upon proliferation arrest predicts individual yeast cell fate. eLife.

[20] Swygert S.G., Kim S., Wu X., Fu T., Hsieh T.H., Rando O.J., Eisenman R.N., Shendure J., McKnight J.N., Tsukiyama T. (2019). Condensin-Dependent Chromatin Compaction Represses Transcription Globally during Quiescence. Mol. Cell.

[21] Guidi M., Ruault M., Marbouty M., Loïodice I., Cournac A., Billaudeau C., Hocher A., Mozziconacci J., Koszul R., Taddei A. (2015). Spatial reorganization of telomeres in long-lived quiescent cells. Genome Biol..

[22] Laporte D., Courtout F., Tollis S., Sagot I. (2016). Quiescent *Saccharomyces cerevisiae* forms telomere hyperclusters at the nuclear membrane vicinity through a multifaceted mechanism involving Esc1, the Sir complex, and chromatin condensation. Mol. Biol. Cell.

[23] Laporte D., Jimenez L., Gouleme L., Sagot I. (2018). Yeast quiescence exit swiftness is influenced by cell volume and chronological age. Microb. Cell.

[24] Bojsen R., Regenberg B., Folkesson A. (2014). *Saccharomyces cerevisiae* biofilm tolerance towards systemic antifungals depends on growth phase. BMC Microbiol..

[25] Opalek M., Smug B., Doebeli M., Wloch-Salamon D. (2022). On the Ecological Significance of Phenotypic Heterogeneity in Microbial Populations Undergoing Starvation. Microbiol. Spectr..

[26] Foo Y.Z., O’Dea R.E., Koricheva J., Nakagawa S., Lagisz M. (2021). A practical guide to question formation, systematic searching and study screening for literature reviews in ecology and evolution. Methods Ecol. Evol..

[27] O’Dea R.E., Lagisz M., Jennions M.D., Koricheva J., Noble D.W.A., Parker T.H., Gurevitch J., Page M.J., Stewart G., Moher D. (2021). Preferred reporting items for systematic reviews and meta-analyses in ecology and evolutionary biology: A PRISMA extension. Biol. Rev..

[28] R Core Team R: A Language and Environment for Statistical Computing 2021. https://www.r-project.org/.

[29] Wickham H., François R., Henry L., Müller K. Dplyr: A Grammar of Data Manipulation 2022. https://cran.r-project.org/package=dplyr.

[30] Wickham H., Bryan J. Readxl: Read Excel Files 2022. https://cran.r-project.org/package=readxl.

[31] Wickham H. Ggplot2: Elegant Graphics for Data Analysis 2016. https://ggplot2.tidyverse.org.

[32] Koneswarakantha B. Easyalluvial: Generate Alluvial Plots with a Single Line of Code 2022. https://github.com/erblast/easyalluvial/.

[33] Inkscape Project Inkscape 2020. https://inkscape.org/.

[34] Lang D., Chien G. Package ‘wordcloud2′: Create Word Cloud by “htmlwidget” 2022. https://github.com/lchiffon/wordcloud2.

[35] An Z., Tassa A., Thomas C., Zhong R., Xiao G., Fotedar R., Tu B.P., Klionsky D.J., Levine B. (2014). Autophagy is required for G1/G0quiescence in response to nitrogen starvation in *Saccharomyces cerevisiae*. Autophagy.

[36] Nussbaum I., Weindling E., Jubran R., Cohen A., Bar-Nun S. (2014). Deteriorated Stress Response in Stationary-Phase Yeast: Sir2 and Yap1 Are Essential for Hsf1 Activation by Heat Shock and Oxidative Stress, Respectively. PLoS ONE.

[37] Garay E., Campos S.E., González de la Cruz J., Gaspar A.P., Jinich A., DeLuna A. (2014). High-Resolution Profiling of Stationary-Phase Survival Reveals Yeast Longevity Factors and Their Genetic Interactions. PLoS Genet..

[38] Mews P., Zee B.M., Liu S., Donahue G., Garcia B.A., Berger S.L. (2014). Histone Methylation Has Dynamics Distinct from Those of Histone Acetylation in Cell Cycle Reentry from Quiescence. Mol. Cell. Biol..

[39] Porzoor A., Caine J.M., Macreadie I.G. (2014). Pretreatment of chemically-synthesized Aβ42 affects its biological activity in yeast. Prion.

[40] Shah K.H., Nostramo R., Zhang B., Varia S.N., Klett B.M., Herman P.K. (2014). Protein kinases are associated with multiple, distinct cytoplasmic granules in quiescent yeast cells. Genetics.

[41] Martins D., English A.M. (2014). SOD1 oxidation and formation of soluble aggregates in yeast: Relevance to sporadic ALS development. Redox Biol..

[42] Acker J., Nguyen N.T.T., Vamme M., Tavenet A., Bri-Suleau A., Conesa C. (2014). Sub1 and Maf1, two effectors of RNA polymerase III, are involved in the yeast quiescence cycle. PLoS ONE.

[43] Sarkar S., Dalgaard J.Z., Millar J.B.A., Arumugam P. (2014). The Rim15-Endosulfine-PP2ACdc55 Signalling Module Regulates Entry into Gametogenesis and Quiescence via Distinct Mechanisms in Budding Yeast. PLoS Genet..

[44] Quan Z., Cao L., Tang Y., Yan Y., Oliver S.G., Zhang N. (2015). The Yeast GSK-3 Homologue Mck1 Is a Key Controller of Quiescence Entry and Chronological Lifespan. PLoS Genet..

[45] Li L., Miles S., Breeden L.L. (2015). A Genetic Screen for *Saccharomyces cerevisiae* Mutants That Fail to Enter Quiescence. G3 Genes Genomes Genet..

[46] Park Y., Han G.S., Mileykovskaya E., Garrett T.A., Carman G.M. (2015). Altered lipid synthesis by lack of yeast Pah1 phosphatidate phosphatase reduces chronological life span. J. Biol. Chem..

[47] Carbó R., Ginovart M., Carta A., Portell X., del Valle L.J. (2015). Effect of aerobic and microaerophilic culture in the growth dynamics of *Saccharomyces cerevisiae* and in training of quiescent and non-quiescent subpopulations. Arch. Microbiol..

[48] Wanichthanarak K., Wongtosrad N., Petranovic D. (2015). Genome-wide expression analyses of the stationary phase model of ageing in yeast. Mech. Ageing Dev..

[49] McKnight J.N., Boerma J.W., Breeden L.L., Tsukiyama T. (2015). Global Promoter Targeting of a Conserved Lysine Deacetylase for Transcriptional Shutoff during Quiescence Entry. Mol. Cell.

[50] Hughes Hallett J.E., Luo X., Capaldi A.P. (2015). Snf1/AMPK promotes the formation of Kog1/raptor-bodies to increase the activation threshold of TORC1 in budding yeast. eLife.

[51] Müller M., Schmidt O., Angelova M., Faserl K., Weys S., Kremser L., Pfaffenwimmer T., Dalik T., Kraft C., Trajanoski Z. (2015). The coordinated action of the MVB pathway and autophagy ensures cell survival during starvation. eLife.

[52] Vasicova P., Lejskova R., Malcova I., Hasek J. (2015). The Stationary-Phase Cells of *Saccharomyces cerevisiae* Display Dynamic Actin Filaments Required for Processes Extending Chronological Life Span. Mol. Cell. Biol..

[53] Rutledge M.T., Russo M., Belton J.M., Dekker J., Broach J.R. (2015). The yeast genome undergoes significant topological reorganization in quiescence. Nucleic Acids Res..

[54] Sundaram V., Petkova M.I., Pujol-Carrion N., Boada J., de la Torre-Ruiz M.A. (2015). Tor1, Sch9 and PKA downregulation in quiescence rely on Mtl1 to preserve mitochondrial integrity and cell survival. Mol. Microbiol..

[55] Boucherie H. (1985). Protein synthesis during transition and stationary phases under glucose limitation in *Saccharomyces cerevisiae*. J. Bacteriol..

[56] Munder M.C., Midtvedt D., Franzmann T., Nüske E., Otto O., Herbig M., Ulbricht E., Müller P., Taubenberger A., Maharana S. (2016). A pH-driven transition of the cytoplasm from a fluid- to a solid-like state promotes entry into dormancy. eLife.

[57] Cao L., Tang Y., Quan Z., Zhang Z., Oliver S.G., Zhang N. (2016). Chronological Lifespan in Yeast Is Dependent on the Accumulation of Storage Carbohydrates Mediated by Yak1, Mck1 and Rim15 Kinases. PLoS Genet..

[58] Oomuro M., Kato T., Zhou Y., Watanabe D., Motoyama Y., Yamagishi H., Akao T., Aizawa M. (2016). Defective quiescence entry promotes the fermentation performance of bottom-fermenting brewer’s yeast. J. Biosci. Bioeng..

[59] Bisschops M.M.M., Luttik M.A.H., Doerr A., Verheijen P.J.T., Bruggeman F., Pronk J.T., Daran-Lapujade P. (2017). Extreme calorie restriction in yeast retentostats induces uniform non-quiescent growth arrest. Biochim. Biophys. Acta-Mol. Cell Res..

[60] Klukovich R., Courchesne W.E. (2016). Functions of *Saccharomyces cerevisiae* Ecm27p, a putative Na^+^/Ca^2+^ exchanger, in calcium homeostasis, carbohydrate storage and cell cycle reentry from the quiescent phase. Microbiol. Res..

[61] Miles S., Croxford M.W., Abeysinghe A.P., Breeden L.L. (2016). Msa1 and Msa2 Modulate G1-Specific Transcription to Promote G1 Arrest and the Transition to Quiescence in Budding Yeast. PLoS Genet..

[62] Kumar R., Srivastava S. (2016). Quantitative proteomic comparison of stationary/G 0 phase cells and tetrads in budding yeast. Sci. Rep..

[63] Svenkrtova A., Belicova L., Volejnikova A., Sigler K., Jazwinski S.M., Pichova A. (2016). Stratification of yeast cells during chronological aging by size points to the role of trehalose in cell vitality. Biogerontology.

[64] Nostramo R., Varia S.N., Zhang B., Emerson M.M., Herman P.K. (2016). The Catalytic Activity of the Ubp3 Deubiquitinating Protease Is Required for Efficient Stress Granule Assembly in *Saccharomyces cerevisiae*. Mol. Cell. Biol..

[65] Wang K., Melki R., Kabani M. (2017). A prolonged chronological lifespan is an unexpected benefit of the [PSI+] prion in yeast. PLoS ONE.

[66] Saul D.J., Walton E.F., Sudbery P.E., Carter B.L.A. (1985). *Saccharomyces cerevisiae* whi2 mutants in stationary phase retain the properties of exponentially growing cells. J. Gen. Microbiol..

[67] Wloch-Salamon D.M., Tomala K., Aggeli D., Dunn B. (2017). Adaptive roles of SSY1 and SIR3 during cycles of growth and starvation in saccharomyces cerevisiae populations enriched for quiescent or nonquiescent cells. G3 Genes Genomes Genet..

[68] Leonov A., Feldman R., Piano A., Arlia-Ciommo A., Lutchman V., Ahmadi M., Elsaser S., Fakim H., Heshmati-Moghaddam M., Hussain A. (2017). Caloric restriction extends yeast chronological lifespan via a mechanism linking cellular aging to cell cycle regulation, maintenance of a quiescent state, entry into a non-quiescent state and survival in the non-quiescent state. Oncotarget.

[69] Robinson C.A., Denison C., Burkenstock A., Nutter C., Gordon D.M. (2017). Cellular conditions that modulate the fungicidal activity of occidiofungin. J. Appl. Microbiol..

[70] Quasem I., Luby C., Mace C., Fuchs S. (2017). Density separation of quiescent yeast using iodixanol. Biotechniques.

[71] Young C.P., Hillyer C., Hokamp K., Fitzpatrick D.J., Konstantinov N.K., Welty J.S., Ness S.A., Werner-Washburne M., Fleming A.B., Osley M.A. (2017). Distinct histone methylation and transcription profiles are established during the development of cellular quiescence in yeast. BMC Genom..

[72] Narayanan V., Schelin J., Gorwa-Grauslund M., Van Niel E.W.J., Carlquist M. (2017). Increased lignocellulosic inhibitor tolerance of *Saccharomyces cerevisiae* cell populations in early stationary phase. Biotechnol. Biofuels.

[73] Kuang Z., Pinglay S., Ji H., Boeke J.D. (2017). Msn2/4 regulate expression of glycolytic enzymes and control transition from quiescence to growth. eLife.

[74] McCleary D.F., Rine J. (2017). Nutritional control of chronological aging and heterochromatin in *Saccharomyces cerevisiae*. Genetics.

[75] Fazal Z., Pelowitz J., Johnson P.E., Harper J.C., Brinker C.J., Jakobsson E. (2017). Three-Dimensional Encapsulation of *Saccharomyces cerevisiae* in Silicate Matrices Creates Distinct Metabolic States as Revealed by Gene Chip Analysis. ACS Nano.

[76] Gu Z.C., Wu E., Sailer C., Jando J., Styles E., Eisenkolb I., Kuschel M., Bitschar K., Wang X., Huang L. (2017). Ubiquitin orchestrates proteasome dynamics between proliferation and quiescence in yeast. Mol. Biol. Cell.

[77] Plesset J., Ludwig J.R., Cox B.S., McLaughlin C.S. (1987). Effect of cell cycle position on thermotolerance in *Saccharomyces cerevisiae*. J. Bacteriol..

[78] Krishna S., Laxman S., Lew D.J. (2018). A minimal “push–pull” bistability model explains oscillations between quiescent and proliferative cell states. Mol. Biol. Cell.

[79] Baroni M.D., Colombo S., Martegani E. (2018). Antagonism between salicylate and the cAMP signal controls yeast cell survival and growth recovery from quiescence. Microb. Cell.

[80] Nevers A., Doyen A., Malabat C., Néron B., Kergrohen T., Jacquier A., Badis G. (2018). Antisense transcriptional interference mediates condition-specific gene repression in budding yeast. Nucleic Acids Res..

[81] Argüello-Miranda O., Liu Y., Wood N.E., Kositangool P., Doncic A. (2018). Integration of Multiple Metabolic Signals Determines Cell Fate Prior to Commitment. Mol. Cell.

[82] Ross E.M., Maxwell P.H. (2018). Low doses of DNA damaging agents extend *Saccharomyces cerevisiae* chronological lifespan by promoting entry into quiescence. Exp. Gerontol..

[83] Lee H.Y., Chao J.C., Cheng K.Y., Leu J.Y. (2018). Misfolding-prone proteins are reversibly sequestered to an Hsp42-associated granule upon chronological aging. J. Cell Sci..

[84] Becker-Kettern J., Paczia N., Conrotte J.F., Zhu C., Fiehn O., Jung P.P., Steinmetz L.M., Linster C.L. (2018). NAD(P)HX repair deficiency causes central metabolic perturbations in yeast and human cells. FEBS J..

[85] Peifer A.C., Maxwell P.H. (2018). Preferential Ty1 retromobility in mother cells and nonquiescent stationary phase cells is associated with increased concentrations of total Gag or processed Gag and is inhibited by exposure to a high concentration of calcium. Aging (Albany NY).

[86] Maqani N., Fine R.D., Shahid M., Li M., Enriquez-Hesles E., Smith J.S. (2018). Spontaneous mutations in CYC8 and MIG1 suppress the short chronological lifespan of budding yeast lacking SNF1/AMPK. Microb. Cell.

[87] Tomova A.A., Kujumdzieva A.V., Petrova V.Y. (2019). Carbon source influences *Saccharomyces cerevisiae* yeast cell survival strategies: Quiescence or sporulation. Biotechnol. Biotechnol. Equip..

[88] Seufert W., McGrath J.P., Jentsch S. (1990). UBC1 encodes a novel member of an essential subfamily of yeast ubiquitin-conjugating enzymes involved in protein degradation. EMBO J..

[89] Cho J.E., Jinks-Robertson S. (2019). Deletions associated with stabilization of the Top1 cleavage complex in yeast are products of the nonhomologous end-joining pathway. Proc. Natl. Acad. Sci. USA.

[90] Zhang J., Martinez-Gomez K., Heinzle E., Wahl S.A. (2019). Metabolic switches from quiescence to growth in synchronized *Saccharomyces cerevisiae*. Metabolomics.

[91] Miles S., Li L.H., Melville Z., Breeden L.L. (2019). Ssd1 and the cell wall integrity pathway promote entry, maintenance, and recovery from quiescence in budding yeast. Mol. Biol. Cell.

[92] Bramasole L., Sinha A., Harshuk D., Cirigliano A., Gurevich S., Yu Z., Carmeli R.L., Glickman M.H., Rinaldi T., Pick E. (2019). The proteasome lid triggers COP9 signalosome activity during the transition of *Sachharomyces cerevisiae* cells into quiescence. Biomolecules.

[93] Barraza C.E., Solari C.A., Rinaldi J., Ojeda L., Rossi S., Ashe M.P., Portela P. (2021). A prion-like domain of Tpk2 catalytic subunit of protein kinase A modulates P-body formation in response to stress in budding yeast. Biochim. Biophys. Acta-Mol. Cell Res..

[94] Shang X., Cao G., Gao H., Li M., Peng G., Ji Y., Zhang Y., Zhang W., Li W., Dou F. (2020). A Single Site Phosphorylation on Hsp82 Ensures Cell Survival during Starvation in *Saccharomyces cerevisiae*. J. Mol. Biol..

[95] Mostofa M.G., Morshed S., Mase S., Hosoyamada S., Kobayashi T., Ushimaru T. (2021). Cdc14 protein phosphatase and topoisomerase II mediate rDNA dynamics and nucleophagic degradation of nucleolar proteins after TORC1 inactivation. Cell. Signal..

[96] Yang R., Bogdan P. (2020). Controlling the Multifractal Generating Measures of Complex Networks. Sci. Rep..

[97] Sun S., Baryshnikova A., Brandt N., Gresham D. (2020). Genetic interaction profiles of regulatory kinases differ between environmental conditions and cellular states. Mol. Syst. Biol..

[98] Marek A., Opalek M., Kałdon A., Mickowska B., Wloch-Salamon D. (2021). Hypersensitive SSY1 mutations negatively influence transition to quiescence in yeast *Saccharomyces cerevisiae*. Yeast.

[99] Poon P.P., Storms R.K. (1991). The periodically expressed TMP1 gene of saccharomyces cerevisiae is subject to START-dependent and START-independent regulation. J. Biol. Chem..

[100] Baroni M.D., Colombo S., Libens O., Pallavi R., Giorgio M., Martegani E., In S. (2020). cerevisiae hydroxycitric acid antagonizes chronological aging and apoptosis regardless of citrate lyase. Apoptosis.

[101] Poramba-Liyanage D.W., Korthout T., Cucinotta C.E., van Kruijsbergen I., van Welsem T., El Atmioui D., Ovaa H., Tsukiyama T., van Leeuwen F. (2020). Inhibition of transcription leads to rewiring of locus-specific chromatin proteomes. Genome Res..

[102] Barré B.P., Hallin J., Yue J.X., Persson K., Mikhalev E., Irizar A., Holt S., Thompson D., Molin M., Warringer J. (2020). Intragenic repeat expansion in the cell wall protein gene HPF1 controls yeast chronological aging. Genome Res..

[103] Kwon Y.Y., Kim S.S., Lee H.J., Sheen S.H., Kim K.H., Lee C.K. (2020). Long-living budding yeast cell subpopulation induced by ethanol/acetate and respiration. Journals Gerontol.-Ser. A Biol. Sci. Med. Sci..

[104] Wood N.E., Kositangool P., Hariri H., Marchand A.J., Henne W.M. (2020). Nutrient Signaling, Stress Response, and Inter-organelle Communication Are Non-canonical Determinants of Cell Fate. Cell Rep..

[105] Long L.J., Lee P.-H., Small E.M., Hillyer C., Guo Y., Osley M.A. (2020). Regulation of UV damage repair in quiescent yeast cells. DNA Repair.

[106] Montella-Manuel S., Pujol-Carrion N., Mechoud M.A., de la Torre-Ruiz M.A. (2021). Bulk autophagy induction and life extension is achieved when iron is the only limited nutrient in *Saccharomyces cerevisiae*. Biochem. J..

[107] Mohammad K., Orfanos E., Titorenko V.I. (2021). Caloric restriction causes a distinct reorganization of the lipidome in quiescent and non-quiescent cells of budding yeast. Oncotarget.

[108] Argüello-Miranda O., Marchand A.J., Kennedy T., Russo M.A.X., Noh J. (2021). Cell cycle–independent integration of stress signals by Xbp1 promotes Non-G1/G0 quiescence entry. J. Cell Biol..

[109] Lee P.H., Osley M.A. (2021). Chromatin structure restricts origin utilization when quiescent cells re-enter the cell cycle. Nucleic Acids Res..

[110] Nurse P., Broek D. (1993). Yeast Cells Can Enter a Quiescent State through G, S, G2, or M Phase of the Cell Cycle. Cancer Res..

[111] Wang R., Huang A., Wang Y., Mei P., Zhu H., Chen Q., Xu S. (2021). High-Resolution Microscopy to Learn the Nuclear Organization of the Living Yeast Cells. Stem Cells Int..

[112] Nicastro R., Raucci S., Michel A.H., Stumpe M., Osuna G.M.G., Jaquenoud M., Kornmann B., de Virgilio C. (2021). Indole-3-acetic acid is a physiological inhibitor of TORC1 in yeast. PLoS Genet..

[113] Daskalova A., Petrova V., Velkova L., Kujumdzieva A., Tomova A., Voelter W., Dolashka P. (2021). Investigation of protein expression of *Saccharomyces cerevisiae* cells in quiescent and proliferating state before and after toxic stress. Biotechnol. Biotechnol. Equip..

[114] Swygert S.G., Lin D., Portillo-Ledesma S., Lin P.Y., Hunt D.R., Kao C.F., Schlick T., Noble W.S., Tsukiyama T. (2021). Local chromatin fiber folding represses transcription and loop extrusion in quiescent cells. eLife.

[115] Daskalova A.V., Tomova A.A., Kujumdzieva A.V., Velkova L.G., Dolashka P.A., Petrova V.Y. (2021). Menadione and hydrogen peroxide trigger specific alterations in RNA polymerases profiles in quiescent *Saccharomyces cerevisiae* cells. Biotechnol. Biotechnol. Equip..

[116] Lesage E., Perez-Fernandez J., Queille S., Dez C., Gadal O., Kwapisz M. (2021). Non-Coding, RNAPII-Dependent Transcription at the Promoters of rRNA Genes Regulates Their Chromatin State in S. cerevisiae. Non-Coding RNA.

[117] Ebrahimi M., Habernig L., Broeskamp F., Aufschnaiter A., Diessl J., Atienza I., Matz S., Ruiz F.A., Büttner S. (2021). Phosphate restriction promotes longevity via activation of autophagy and the multivesicular body pathway. Cells.

[118] Dokládal L., Stumpe M., Hu Z., Jaquenoud M., Dengjel J., De Virgilio C. (2021). Phosphoproteomic responses of TORC1 target kinases reveal discrete and convergent mechanisms that orchestrate the quiescence program in yeast. Cell Rep..

[119] Neil A.J., Hisey J.A., Quasem I., McGinty R.J., Hitczenko M., Khristich A.N., Mirkin S.M. (2021). Replication-independent instability of Friedreich’s ataxia GAA repeats during chronological aging. Proc. Natl. Acad. Sci. USA.

[120] Cucinotta C.E., Dell R.H., Braceros K.C.A., Tsukiyama T. (2021). RSC primes the quiescent genome for hypertranscription upon cell-cycle re-entry. eLife.

[121] Lennon K., Pretel R., Kesselheim J., Hessen S.T., Kukuruzinska M.A. (1995). Proliferation-dependent differential regulation of the dolichol pathway genes in saccharomyces cerevisiae. Glycobiology.

[122] Lim S., Ahn H., Duan R., Liu Y., Ryu H.Y., Ahn S.H. (2021). The Spt7 subunit of the SAGA complex is required for the regulation of lifespan in both dividing and nondividing yeast cells. Mech. Ageing Dev..

[123] Mendoza M., Egervari G., Sidoli S., Donahue G., Alexander D.C., Sen P., Garcia B.A., Berger S.L. (2022). Enzymatic transfer of acetate on histones from lysine reservoir sites to lysine activating sites. Sci. Adv..

[124] Pisareva E.I., Tomova A.A., Petrova V.Y. (2021). *Saccharomyces cerevisiae* quiescent cells: Cadmium resistance and adaptive response. Biotechnol. Biotechnol. Equip..

[125] Bailey T.B., Whitty P.A., Selker E.U., McKnight J.N., McKnight L.E. (2022). Tup1 is critical for transcriptional repression in Quiescence in S. cerevisiae. PLoS Genet..

[126] Breeden L., Miles S. (2022). A common SSD1 truncation is toxic to cells entering quiescence and promotes sporulation. Micropublication Biol..

[127] Cesur M.F., Çakır T., Pir P. (2022). Genome-Wide Analysis of Yeast Metabolic Cycle through Metabolic Network Models Reveals Superiority of Integrated ATAC-seq Data over RNA-seq Data. mSystems.

[128] Galkina K.V., Zubareva V.M., Kashko N.D., Lapashina A.S., Markova O.V., Feniouk B.A., Knorre D.A. (2022). Heterogeneity of Starved Yeast Cells in IF1 Levels Suggests the Role of This Protein in vivo. Front. Microbiol..

[129] Irvali D., Schlottmann F.P., Muralidhara P., Nadelson I., Kleemann K., Wood N.E., Doncic A., Ewald J.C. (2023). When yeast cells change their mind: Cell cycle “Start” is reversible under starvation. EMBO J..

[130] Leonov A., Feldman R., Piano A., Arlia-Ciommo A., Junio J.A.B., Orfanos E., Tafakori T., Lutchman V., Mohammad K., Elsaser S. (2022). Diverse geroprotectors differently affect a mechanism linking cellular aging to cellular quiescence in budding yeast. Oncotarget.

[131] Marinovska P.G., Todorova T.I., Boyadzhiev K.P., Pisareva E.I., Tomova A.A., Parvanova P.N., Dimitrova M., Chankova S.G., Petrova V.Y. (2022). Cellular susceptibility and oxidative stress response to menadione of logarithmic, quiescent, and nonquiescent *Saccharomyces cerevisiae* cell populations. BioRisk.

[132] Ohtani K., DeGregori J., Leone G., Herendeen D.R., Kelly T.J., Nevins J.R. (1996). Expression of the HsOrc1 gene, a human ORC1 homolog, is regulated by cell proliferation via the E2F transcription factor. Mol. Cell. Biol..

[133] Marinovska P., Todorova T., Tomova A., Pisareva E., Boyadzhiev K., Dimitrov M., Parvanova P., Todorova M., Chankova S., Petrova V. (2022). *Saccharomyces cerevisiae* yeast cells as a test system for assessing Zeocin toxicity. BioRisk.

[134] Miles S., Breeden L.L. (2022). Whi7/Srl3 polymorphisms reveal its role in cell size and quiescence. microPublication Biol..

[135] Miyata N., Ito T., Nakashima M., Fujii S., Kuge O. (2022). Mitochondrial phosphatidylethanolamine synthesis affects mitochondrial energy metabolism and quiescence entry through attenuation of Snf1/AMPK signaling in yeast. FASEB J..

[136] Peselj C., Ebrahimi M., Broeskamp F., Prokisch S., Habernig L., Alvarez-Guerra I., Kohler V., Vögtle F.N., Büttner S. (2022). Sterol Metabolism Differentially Contributes to Maintenance and Exit of Quiescence. Front. Cell Dev. Biol..

[137] Willis S.D., Hanley S.E., Doyle S.J., Beluch K., Strich R., Cooper K.F. (2022). Cyclin C-Cdk8 Kinase Phosphorylation of Rim15 Prevents the Aberrant Activation of Stress Response Genes. Front. Cell Dev. Biol..

[138] Zhang Z., Zhang G.R. (2022). Chromosome-condensed G1 phase yeast cells are tolerant to desiccation stress. Microb. Cell.

[139] Jiang Y., Davis C., Broach J.R. (1998). Efficient transition to growth on fermentable carbon sources in *Saccharomyces cerevisiae* requires signaling through the Ras pathway. EMBO J..

[140] Paz I., Meunier J.R., Choder M. (1999). Monitoring dynamics of gene expression in yeast during stationary phase. Gene.

[141] McHugh P.J., Sones W.R., Hartley J.A. (2000). Repair of Intermediate Structures Produced at DNA Interstrand Cross-Links in *Saccharomyces cerevisiae*. Mol. Cell. Biol..

[142] Paz I., Choder M. (2001). Eukaryotic translation initiation factor 4E-dependent translation is not essential for survival of starved yeast cells. J. Bacteriol..

[143] Krause S.A., Gray J.V. (2002). The protein kinase C pathway is required for viability in quiescence in *Saccharomyces cerevisiae*. Curr. Biol..

[144] Werner-Washburne M., Wylie B., Boyack K., Fuge E., Galbraith J., Weber J., Davidson G. (2002). Comparative analysis of multiple genome-scale data sets. Genome Res..

[145] Piper P.W., Bringloe D. (2002). Loss of prohibitins, though it shortens the replicative life span of yeast cells undergoing division, does not shorten the chronological life span of G0-arrested cells. Mech. Ageing Dev..

[146] Sousa-Lopes A., Antunes F., Cyrne L., Marinho H.S. (2004). Decreased cellular permeability to H 2O 2 protects *Saccharomyces cerevisiae* cells in stationary phase against oxidative stress. FEBS Lett..

[147] Hossain M.S., Kurokawa K., Akimitsu N., Sekimizu K. (2004). DNA topoisomerase II is required for the G0-to-S phase transition in Drosophila Schneider cells, but not in yeast. Genes to Cells.

[148] Martinez M.J., Roy S., Archuletta A.B., Wentzell P.D., Anna-Arriola S.S., Rodriguez A.L., Aragon A.D., Quiñones G.A., Allen C., Werner-Washburne M. (2004). Genomic Analysis of Stationary-Phase and Exit in *Saccharomyces cerevisiae*: Gene Expression and Identification of Novel Essential Genes. Mol. Biol. Cell.

[149] Frankenberg-Schwager M., Kirchermeier D., Greif G., Baer K., Becker M., Frankenberg D. (2005). Cisplatin-mediated DNA double-strand breaks in replicating but not in quiescent cells of the yeast *Saccharomyces cerevisiae*. Toxicology.

[150] Sagot I., Schaeffer J., Daignan-Fornier B. (2005). Guanylic nucleotide starvation affects *Saccharomyces cerevisiae* mother-daughter separation and may be a signal for entry into quiescence. BMC Cell Biol..

[151] Radonjic M., Andrau J.C., Lijnzaad P., Kemmeren P., Kockelkorn T.T.J.P., Van Leenen D., Van Berkum N.L., Holstege F.C.P. (2005). Genome-wide analyses reveal RNA polymerase II located upstream of genes poised for rapid response upon *S. cerevisiae* stationary phase exit. Mol. Cell.

[152] Wanke V., Pedruzzi I., Cameroni E., Dubouloz F., De Virgilio C. (2005). Regulation of G0 entry by the Pho80-Pho85 cyclin-CDK complex. EMBO J..

[153] Dubouloz F., Deloche O., Wanke V., Cameroni E., De Virgilio C. (2005). The TOR and EGO protein complexes orchestrate microautophagy in yeast. Mol. Cell.

[154] Sagot I., Pinson B., Salin B., Daignan-Fornier B. (2006). Actin Bodies in Yeast Quiescent Cells: An Immediately Available Actin Reserve?. Mol. Biol. Cell.

[155] Escusa S., Camblong J., Galan J.M., Pinson B., Daignan-Fornier B. (2006). Proteasome- and SCF-dependent degradation of yeast adenine deaminase upon transition from proliferation to quiescence requires a new F-box protein named Saf1p. Mol. Microbiol..

[156] Aragon A.D., Quiñones G.A., Thomas E.V., Roy S., Werner-Washburne M. (2006). Release of extraction-resistant mRNA in stationary phase *Saccharomyces cerevisiae* produces a massive increase in transcript abundance in response to stress. Genome Biol..

[157] Slattery M.G., Heideman W. (2007). Coordinated regulation of growth genes in *Saccharomyces cerevisiae*. Cell Cycle.

[158] Weinberger M., Feng L., Paul A., Smith D.L., Hontz R.D., Smith J.S., Vujcic M., Singh K.K., Huberman J.A., Burhans W.C. (2007). DNA replication stress is a determinant of chronological lifespan in budding yeast. PLoS ONE.

[159] Gomes P., Sampaio-Marques B., Ludovico P., Rodrigues F., Leão C. (2007). Low auxotrophy-complementing amino acid concentrations reduce yeast chronological life span. Mech. Ageing Dev..

[160] Escusa S., Laporte D., Massoni A., Boucherie H., Dautant A., Daignan-Fornier B. (2007). Skp1-Cullin-F-box-dependent degradation of Aah1p requires its interaction with the F-box protein Saf1p. J. Biol. Chem..

[161] Liko D., Slattery M.G., Heideman W. (2007). Stb3 binds to ribosomal RNA processing element motifs that control transcriptional responses to growth in *Saccharomyces cerevisiae*. J. Biol. Chem..

[162] Murakami C.J., Burtner C.R., Kennedy B.K., Kaeberlein M. (2008). A method for high-throughput quantitative analysis of yeast chronological life span. J. Gerontol.-Ser. A Biol. Sci. Med. Sci..

[163] Aragon A.D., Rodriguez A.L., Meirelles O., Roy S., Davidson G.S., Tapia P.H., Allen C., Joe R., Benn D., Werner-Washburne M. (2008). Characterization of Differentiated Quiescent and Nonquiescent Cells in Yeast Stationary-Phase Cultures. Mol. Biol. Cell.

[164] Sahin A., Daignan-Fornier B., Sagot I. (2008). Polarized growth in the absence of F-actin in *Saccharomyces cerevisiae* exiting quiescence. PLoS ONE.

[165] Laporte D., Salin B., Daignan-Fornier B., Sagot I. (2008). Reversible cytoplasmic localization of the proteasome in quiescent yeast cells. J. Cell Biol..

[166] Friis R.M.N., Wu B.P., Reinke S.N., Hockman D.J., Sykes B.D., Schultz M.C. (2009). A glycolytic burst drives glucose induction of global histone acetylation by picNuA4 and SAGA. Nucleic Acids Res..

[167] Barea F., Bonatto D. (2009). Aging defined by a chronologic-replicative protein network in *Saccharomyces cerevisiae*: An interactome analysis. Mech. Ageing Dev..

[168] Li L., Lu Y., Qin L.-X., Bar-Joseph Z., Werner-Washburne M., Breeden L.L. (2009). Budding Yeast SSD1-V Regulates Transcript Levels of Many Longevity Genes and Extends Chronological Life Span in Purified Quiescent Cells. Mol. Biol. Cell.

[169] Goldberg A.A., Bourque S.D., Kyryakov P., Gregg C., Boukh-Viner T., Beach A., Burstein M.T., Machkalyan G., Richard V., Rampersad S. (2009). Effect of calorie restriction on the metabolic history of chronologically aging yeast. Exp. Gerontol..

[170] Benbadis L., Cot M., Rigoulet M., Francois J. (2009). Isolation of two cell populations from yeast during high-level alcoholic fermentation that resemble quiescent and nonquiescent cells from the stationary phase on glucose. FEMS Yeast Res..

[171] Minois N., Lagona F., Frajnt M., Vaupel J.W. (2009). Plasticity of death rates in stationary phase in *Saccharomyces cerevisiae*. Aging Cell.

[172] Tapia H., Morano K.A. (2010). Hsp90 Nuclear Accumulation in Quiescence Is Linked to Chaperone Function and Spore Development in Yeast. Mol. Biol. Cell.

[173] Fabrizio P., Hoon S., Shamalnasab M., Galbani A., Wei M., Giaever G., Nislow C., Longo V.D. (2010). Genome-wide screen in *Saccharomyces cerevisiae* identifies vacuolar protein sorting, autophagy, biosynthetic, and tRNA methylation genes involved in life span regulation. PLoS Genet..

[174] Weinberger M., Mesquita A., Carroll T., Marks L., Yang H., Zhang Z., Ludovico P., Burhans W.C. (2010). Growth signaling promotes chronological aging in budding yeast by inducing superoxide anions that inhibit quiescence. Aging (Albany NY).

[175] Talarek N., Cameroni E., Jaquenoud M., Luo X., Bontron S., Lippman S., Devgan G., Snyder M., Broach J.R., De Virgilio C. (2010). Initiation of the TORC1-Regulated G0 Program Requires Igo1/2, which License Specific mRNAs to Evade Degradation via the 5′-3′ mRNA Decay Pathway. Mol. Cell.

[176] Petkova M.I., Pujol-Carrion N., Arroyo J., García-Cantalejo J., De La Torre-Ruiz M.A. (2010). Mtl1 is required to activate general stress response through TOR1 and RAS2 inhibition under conditions of glucose starvation and oxidative stress. J. Biol. Chem..

[177] Liko D., Conway M.K., Grunwald D.S., Heideman W. (2010). Stb3 plays a role in the glucose-induced transition from quiescence to growth in *Saccharomyces cerevisiae*. Genetics.

[178] Gresham D., Boer V.M., Caudy A., Ziv N., Brandt N.J., Storey J.D., Botstein D. (2011). System-level analysis of genes and functions affecting survival during nutrient starvation in *Saccharomyces cerevisiae*. Genetics.

[179] Vodenicharov M.D., Laterreur N., Wellinger R.J. (2010). Telomere capping in non-dividing yeast cells requires Yku and Rap1. EMBO J..

[180] Emerman A.B., Zhang Z.-R., Chakrabarti O., Hegde R.S. (2010). Trehalose Is a Key Determinant of the Quiescent Metabolic State That Fuels Cell Cycle Progression upon Return to Growth. Mol. Biol. Cell.

[181] Roy S., Werner-Washburne M., Lane T. (2011). A multiple network learning approach to capture system-wide condition-specific responses. Bioinformatics.

[182] Boender L.G.M., van Maris A.J.A., de Hulster E.A.F., Almering M.J.H., van der Klei I.J., Veenhuis M., de Winde J.H., Pronk J.T., Daran-Lapujade P. (2011). Cellular responses of *Saccharomyces cerevisiae* at near-zero growth rates: Transcriptome analysis of anaerobic retentostat cultures. FEMS Yeast Res..

[183] Harper J.C., Lopez D.M., Larkin E.C., Economides M.K., McIntyre S.K., Alam T.M., Tartis M.S., Werner-Washburne M., Brinker C.J., Brozik S.M. (2011). Encapsulation of S. cerevisiae in poly(glycerol) silicate derived matrices: Effect of matrix additives and cell metabolic phase on long-term viability and rate of gene expression. Chem. Mater..

[184] Boender L.G.M., Almering M.J.H., Dijk M., van Maris A.J.A., de Winde J.H., Pronk J.T., Daran-Lapujade P. (2011). Extreme calorie restriction and energy source starvation in *Saccharomyces cerevisiae* represent distinct physiological states. Biochim. Biophys. Acta-Mol. Cell Res..

[185] Luo X., Talarek N., De Virgilio C. (2011). Initiation of the yeast G0 program requires Igo1 and Igo2, which antagonize activation of decapping of specific nutrient-regulated mRNAs. RNA Biol..

[186] Slavov N., Macinskas J., Caudy A., Botstein D. (2011). Metabolic cycling without cell division cycling in respiring yeast. Proc. Natl. Acad. Sci. USA.

[187] Laporte D., Lebaudy A., Sahin A., Pinson B., Ceschin J., Daignan-Fornier B., Sagot I. (2011). Metabolic status rather than cell cycle signals control quiescence entry and exit. J. Cell Biol..

[188] Kelly M.K., Alver B., Kirkpatrick D.T. (2011). Minisatellite alterations in ZRT1 mutants occur via RAD52-dependent and RAD52-independent mechanisms in quiescent stationary phase yeast cells. DNA Repair.

[189] Ngubo M., Kemp G., Patterton H.G. (2011). Nano-electrospray tandem mass spectrometric analysis of the acetylation state of histones H3 and H4 in stationary phase in *Saccharomyces cerevisiae*. BMC Biochem..

[190] Zakrajšek T., Raspor P., Jamnik P. (2011). *Saccharomyces cerevisiae* in the stationary phase as a model organism—characterization at cellular and proteome level. J. Proteomics.

[191] Urbanczyk H., Noguchi C., Wu H., Watanabe D., Akao T., Takagi H., Shimoi H. (2011). Sake yeast strains have difficulty in entering a quiescent state after cell growth cessation. J. Biosci. Bioeng..

[192] Ramachandran V., Shah K.H., Herman P.K. (2011). The cAMP-Dependent Protein Kinase Signaling Pathway Is a Key Regulator of P Body Foci Formation. Mol. Cell.

[193] Davidson G.S., Joe R.M., Roy S., Meirelles O., Allen C.P., Wilson M.R., Tapia P.H., Manzanilla E.E., Dodson A.E., Chakraborty S. (2011). The proteomics of quiescent and nonquiescent cell differentiation in yeast stationary-phase cultures. Mol. Biol. Cell.

[194] Bonzanni N., Zhang N., Oliver S.G., Fisher J. (2011). The role of proteosome-mediated proteolysis in modulating potentially harmful transcription factor activity in *Saccharomyces cerevisiae*. Bioinformatics.

[195] Escoté X., Miranda M., Rodríguez-Porrata B., Mas A., Cordero R., Posas F., Vendrell J. (2011). The stress-activated protein kinase Hog1 develops a critical role after resting state. Mol. Microbiol..

[196] Watanabe D., Araki Y., Zhou Y., Maeya N., Akao T., Shimoi H. (2012). A Loss-of-Function Mutation in the PAS Kinase Rim15p Is Related to Defective Quiescence Entry and High Fermentation Rates of *Saccharomyces cerevisiae* Sake Yeast Strains. Appl. Environ. Microbiol..

[197] Kyryakov P., Beach A., Richard V.R., Burstein M.T., Leonov A., Levy S., Titorenko V.I. (2012). Caloric restriction extends yeast chronological lifespan by altering a pattern of age-related changes in trehalose concentration. Front. Physiol..

[198] Conway M.K., Grunwald D., Heideman W. (2012). Glucose, nitrogen, and phosphate repletion in saccharomyces cerevisiae: Common transcriptional responses to different nutrient signals. G3 Genes Genomes Genet..

[199] Burstein M.T., Kyryakov P., Beach A., Richard V.R., Koupaki O., Gomez-Perez A., Leonov A., Levy S., Noohi F., Titorenko V.I. (2012). Lithocholic acid extends longevity of chronologically aging yeast only if added at certain critical periods of their lifespan. Cell Cycle.

[200] Reimand J., Aun A., Vilo J., Vaquerizas J.M., Sedman J., Luscombe N.M. (2012). M:Explorer: Multinomial regression models reveal positive and negative regulators of longevity in yeast quiescence. Genome Biol..

[201] Petkova M.I., Pujol-Carrion N., de la Torre-Ruiz M.A. (2012). Mtl1 O-mannosylation mediated by both Pmt1 and Pmt2 is important for cell survival under oxidative conditions and TOR blockade. Fungal Genet. Biol..

[202] Kelly M.K., Brosnan L., Jauert P.A., Dunham M.J., Kirkpatrick D.T. (2012). Multiple pathways regulate minisatellite stability during stationary phase in yeast. G3 Genes Genomes Genet..

[203] Murakami C., Delaney J.R., Chou A., Carr D., Schleit J., Sutphin G.L., An E.H., Castanza A.S., Fletcher M., Goswami S. (2012). pH neutralization protects against reduction in replicative lifespan following chronological aging in yeast. Cell Cycle.

[204] Hanna J., Waterman D., Boselli M., Finley D. (2012). Spg5 protein regulates the proteasome in quiescence. J. Biol. Chem..

[205] Liu I.C., Chiu S.W., Lee H.Y., Leu J.Y. (2012). The histone deacetylase Hos2 forms an Hsp42-dependent cytoplasmic granule in quiescent yeast cells. Mol. Biol. Cell.

[206] Alver B., Jauert P.A., Brosnan L., O’Hehir M., VanderSluis B., Myers C.L., Kirkpatrick D.T. (2013). A whole genome screen for minisatellite stability genes in stationary-phase yeast cells. G3 Genes Genomes Genet..

[207] Lei S., Tu B.P. (2013). Acetyl-CoA induces transcription of the key G1 cyclin CLN3 to promote entry into the cell division cycle in *Saccharomyces cerevisiae*. Proc. Natl. Acad. Sci. USA.

[208] Laporte D., Courtout F., Salin B., Ceschin J., Sagot I. (2013). An array of nuclear microtubules reorganizes the budding yeast nucleus during quiescence. J. Cell Biol..

[209] Weinberger M., Sampaio-Marques B., Ludovico P., Burhans W.C. (2013). DNA replication stress-induced loss of reproductive capacity in s. cerevisiae and its inhibition by caloric restriction. Cell Cycle.

[210] Saunier R., Esposito M., Dassa E.P., Delahodde A. (2013). Integrity of the *Saccharomyces cerevisiae* Rpn11 Protein Is Critical for Formation of Proteasome Storage Granules (PSG) and Survival in Stationary Phase. PLoS ONE.

[211] Li L., Miles S., Melville Z., Prasad A., Bradley G., Breeden L.L. (2013). Key events during the transition from rapid growth to quiescence in budding yeast require posttranscriptional regulators. Mol. Biol. Cell.

[212] Casatta N., Porro A., Orlandi I., Brambilla L., Vai M. (2013). Lack of Sir2 increases acetate consumption and decreases extracellular pro-aging factors. Biochim. Biophys. Acta-Mol. Cell Res..

[213] Webb K.J., Xu T., Park S.K., Yates J.R. (2013). Modified MuDPIT Separation Identified 4488 Proteins in a System-wide Analysis of Quiescence in Yeast. J. Proteome Res..

[214] Alver B., Kelly M.K., Kirkpatrick D.T. (2013). Novel Checkpoint Pathway Organization Promotes Genome Stability in Stationary-Phase Yeast Cells. Mol. Cell. Biol..

[215] Shah K.H., Zhang B., Ramachandran V., Herman P.K. (2013). Processing body and stress granule assembly occur by independent and Differentially regulated pathways in *Saccharomyces cerevisiae*. Genetics.

[216] Lee P., Kim M.S., Paik S.M., Choi S.H., Cho B.R., Hahn J.S. (2013). Rim15-dependent activation of Hsf1 and Msn2/4 transcription factors by direct phosphorylation in *Saccharomyces cerevisiae*. FEBS Lett..

[217] Miles S., Li L., Davison J., Breeden L.L. (2013). Xbp1 Directs Global Repression of Budding Yeast Transcription during the Transition to Quiescence and Is Important for the Longevity and Reversibility of the Quiescent State. PLoS Genet..

[218] Bontron S., Jaquenoud M., Vaga S., Talarek N., Bodenmiller B., Aebersold R., De Virgilio C. (2013). Yeast Endosulfines Control Entry into Quiescence and Chronological Life Span by Inhibiting Protein Phosphatase 2A. Cell Rep..

[219] Palkova Z. (2004). Multicellular microorganisms: Laboratory versus nature. EMBO Rep..

[220] Mozzachiodi S., Bai F.Y., Baldrian P., Bell G., Boundy-Mills K., Buzzini P., Čadež N., Cubillos F.A., Dashko S., Dimitrov R. (2022). Yeasts from temperate forests. Yeast.

[221] Finkel S.E., Kolter R. (1999). Evolution of microbial diversity during prolonged starvation. Proc. Natl. Acad. Sci. USA.

[222] Zambrano M.M., Kolter R. (1996). GASPing for Life in Stationary Phase. Cell.

[223] Aouizerat T., Gelman D., Szitenberg A., Gutman I., Glazer S., Reich E., Schoemann M., Kaplan R., Saragovi A., Hazan R. (2019). Eukaryotic Adaptation to Years-Long Starvation Resembles that of Bacteria. iScience.

