# Supplementary material for: A Systematic Review on Quiescent State Research Approaches in S. cerevisiae
Source: Cells. 2023 Jun 12;12(12):1608. doi: 10.3390/cells12121608 (PMC10297366; doi:10.3390/cells12121608)
Supplement: Supplementary file 1 [file cells-12-01608-s001.zip › Qmeth_Supp.M._Cells_2023.05.03.pdf]

SUPPLEMENTARY MATERIALS

Opalek M., *et al* 2023
A systematic review on quiescent state research approaches in *S. cerevisiae*

SEARCH STRINGS

1st search

Databases search on 09.12.2021

Web of Science Number of records: 3 290

TOPIC quiesce* OR “stationary” OR “G0” AND TOPIC "Saccharomyces cerevisiae" OR "S. cerevisiae" OR "yeast" OR "yeasts"

Scopus Number of records: 3 373

(TITLE-ABS-KEY (quiesce* OR "stationary" OR "G0") AND TITLE-ABS-KEY ("Saccharomyces cerevisiae" OR "S. cerevisiae" OR "yeast" OR "yeasts"))

PubMed Number of records: 2 928

(quiesce* OR "stationary" OR "G0") AND ("Saccharomyces cerevisiae" OR "S. cerevisiae" OR "yeast" OR "yeasts")

After deduplication: 4 687 unique records

2nd search

Databases search on 25.01.2022.

Web of Science

Updates of the 1st search Number of records: 15

quiesce* OR “stationary” OR “G0” (Topic) and "Saccharomyces cerevisiae" OR "S. cerevisiae" OR "yeast" OR "yeasts" (Topic) | Timespan: 2021-12-01 to 2022-01-25 (Index Date)

2st search string Number of records: 1,274

#1 NOT #2

#1 dorman* OR (grow* NEAR/4 arrest*) (Topic) and "Saccharomyces cerevisiae" OR "S. cerevisiae" OR "yeast" OR "yeasts" (Topic) and Articles (Document Types) and English (Languages) | number of records: 1,380

#2 quiesce* OR “stationary” OR “G0” (Topic) and "Saccharomyces cerevisiae" OR "S. cerevisiae" OR "yeast" OR "yeasts" (Topic) and Articles (Document Types) and English (Languages) | number of records: 3,289

Scopus

Updates of the 1st search Number of records: 17

(TITLE-ABS-KEY (quiesce* OR "stationary" OR "G0") AND TITLE-ABS-KEY ("Saccharomyces cerevisiae" OR "S. cerevisiae" OR "yeast" OR "yeasts")) AND RECENT (60)

2st search string Number of records: 1 193

((TITLE-ABS-KEY (dorman* OR (grow* W/4 arrest*)) AND TITLE-ABS-KEY ("Saccharomyces cerevisiae" OR "S. cerevisiae" OR yeast OR yeasts))) AND NOT ((TITLE-ABS-KEY (quiesce* OR "stationary" OR "G0") AND TITLE-ABS-KEY ("Saccharomyces cerevisiae" OR "S. cerevisiae" OR yeast OR yeasts)))

PubMed

Updates of the 1st search Number of records: 11

(quiesce* OR "stationary" OR "G0") AND ("Saccharomyces cerevisiae" OR "S. cerevisiae" OR "yeast" OR "yeasts") | Filters applied: from 2021/12/1 - 2022/1/25.

2st search string Number of records: 895

PubMed has no operator ‘with/near’ as such the “grow* WITH arrest*” was replaced by “grow arrest*” OR “growth arrest*” keywords

(("dorman*"[All Fields] OR ("grow"[All Fields] AND "arrest*"[All Fields]) OR "growth arrest*"[All Fields]) AND ("Saccharomyces cerevisiae"[All Fields] OR "S. cerevisiae"[All Fields] OR "yeast"[All Fields] OR "yeasts"[All Fields])) NOT (("quiesce*"[All Fields] OR "stationary"[All Fields] OR "G0"[All Fields]) AND ("Saccharomyces cerevisiae"[All Fields] OR "S. cerevisiae"[All Fields] OR "yeast"[All Fields] OR "yeasts"[All Fields]))

After deduplication 1 774 unique records were added.

3rd search

Web of Science Number of records: 129

((TS=(quiesce* OR “stationary” OR “G0” OR dorman* OR (grow* NEAR/4 arrest*))) AND TS=("Saccharomyces cerevisiae" OR "S. cerevisiae" OR "yeast" OR "yeasts")) AND PY=(2022)

Scopus Number of records: 127

(TITLE-ABS-KEY (quiesce* OR "stationary" OR "G0" OR dorman* OR (grow* W/4 arrest*)) AND TITLE-ABS-KEY ("Saccharomyces cerevisiae" OR "S. cerevisiae" OR "yeast" OR "yeasts")) AND (LIMIT-TO (PUBYEAR , 2022))

PubMed Number of records: 99
((quiesce* OR "stationary" OR "G0" OR "dorman*" OR "grow arrest*" OR "growth arrest*")) AND ("Saccharomyces cerevisiae" OR "S. cerevisiae" OR "yeast" OR "yeasts")
Filters applied: from 2022/1/1 - 2022/12/31

Altogether, after 3rd search, 177 unique records (after deduplication) were added.

Decision tree

The decision tree applied for initial screening of records, based on title, abstract and keywords performed in Rayyan.

1) Was the article written in English?
   - YES/MAYBE → go to (2)
   - NO → EXCLUDE

2) Was the research conducted on *S. cerevisiae*?
   - YES/MAYBE → go to (3)
   - NO → EXCLUDE

3) Does the article present original research?
   - YES/MAYBE → go to (4)
   - NO → EXCLUDE
     EXCLUDE: reviews, chapters, books, methodological articles

4) Was the quiescence/stationary phase/starvation the topic of the research?
   - YES/MAYBE → **INCLUDE**
   - NO → EXCLUDE
     EXCLUDE: “stationary phase” was only the environment/condition in which given tests were conducted or was used to referred to the age of tested population

Reasons for the exclusion of records during the full-text screening phase (205 research articles):

- words “quiescent” or “quiescence” were not used at all or not in the context of results (e.g. quiescence was mentioned in introduction but not investigated)
- wrong article type - e.g. reviews

- research conducted on yeast spores
- research conducted on other organism than *S. cerevisiae*

Please see the Supplementary File for a list of the excluded articles, along with corresponding reason for exclusion.

Details on the categorization used during full-text screening

Please see the Supplementary File for a list of the included articles, along with classification for all analysed features.

In the tables below (Table S1 - S5), there are details on the categories used to analyse articles during the full-text screening. Each table correspond to one aspect/feature of an article that was analysed. For each feature/question, there were pre-defined categories to which article were classified to. All categories, except of metabolic profile, are not exclusive, meaning that one article can be classified to more than one category within given feature.

Table S1. The meaning of quiescence

Category	Description
whole_pop	Whole stationary phase/starved population is assumed to be in quiescent state
subpop	Subpopulations of phenotypically distinct cells are identified and/or separated from stationary phase population

Table S2. The age of studied populations - how long was a population incubated to reach quiescent state/when authors defined quiescence entry

Category	Time
less than 24h	0 – 24 h
24h - 48h	1 - 2 days
49h - 96h	2 - 4 days
4 days - 1 week	+96 h, up to 7 days, 168 h
1 - 2 weeks	+7 days, up to 14 days
more than 14 days	+14 days
not specified / not applicable	

Table S3. How quiescence was induced

Category	Description
gradual_starvation	The population was left in growth media for defined time, or was grown in nutrient-limiting conditions for prolonged time (retinostat)

abrupt_starvation	The population was transferred to starvation media or some growth-arresting factor was added (e.g rapamycin, alpha-factor)

Table S4. The metabolic profile of studied strain

Category	Description
prototrophs	Single prototrophic strain or collection of various prototrophic strains
both	Collection of prototrophic and auxotrophic strains
auxotrophs	Single auxotrophic strain or collection of various auxotrophic strains
not specified	

Table S5. Biological aspects

Category	Description
Life span	Long-term survival, caloric restriction, chronological and replicative life span, viability, apoptosis, reproductive capacity, death rate
Growth phenotypes	Stress response (antifungals, drugs, heat shock), cell-cell, and cell-environment interactions, growth kinetics, oxidative stress, growth curves,
Cell signalling	Cation balance, coordinated cells' answer, signalling pathways (e.g. TOR, MAPK, PKA, HOG pathways) signalling pathway is studied, not the amino acids/proteins themselves, regulatory pathways and networks, proteins interactions, genes interactions
Gene expression	Mechanism and regulation of protein synthesis, ribosome biogenesis, transcriptional networks, translation, RNA-processing, RNA degradation, chromatin and transcription, transcriptional regulation, transcript abundance, protein abundance
Cell cycle	Budding, proliferation entry and exit, cell cycle phases, growth arrests at given cell cycle stage, spores
Genome organization and integrity	Chromosome segregation and folding, chromosome structure, methylation, mechanisms preventing genome instability, DNA damage and repair, mutation rate, histones
Cell's morphology	Descriptive studies on cells' morphology, spatial organization of organelles, bud scars, mitochondrial shape,
Metabolism - Carbon	responses to carbon sources, fermentation and respiration
Metabolism - Storage materials and other	Storage materials - glycolipids, trehalose, autophagy, proteasome, glycogen, ATP, NADPH,
Metabolism - Amino-acids and nutrients	amino-acid, nutrient sensing pathways, responses of cell to nutrient availability, nitrogen, phosphate, amino-acid content
Other	e.g. genetic screening
